# *Entamoeba histolytica* activation of caspase-1 degrades cullin that attenuates NF-κB dependent signaling from macrophages

**DOI:** 10.1371/journal.ppat.1009936

**Published:** 2021-09-09

**Authors:** Attinder Chadha, France Moreau, Shanshan Wang, Antoine Dufour, Kris Chadee

**Affiliations:** 1 Departments of Microbiology, Immunology and Infectious Diseases, University of Calgary, Calgary, Alberta, Canada; 2 Physiology and Pharmacology, University of Calgary, Calgary, Alberta, Canada; 3 Biochemistry and Molecular Biology, Snyder Institute for Chronic Diseases, University of Calgary, Calgary, Alberta, Canada; University of São Paulo FMRP/USP, BRAZIL

## Abstract

While *Entamoeba histolytica* (*Eh*)-induced pro-inflammatory responses are critical in disease pathogenesis, the downstream signaling pathways that subsequently dampens inflammation and the immune response remains unclear. *Eh* in contact with macrophages suppresses NF-κB signaling while favoring NLRP3-dependent pro-inflammatory cytokine production by an unknown mechanism. Cullin-1 and cullin-5 (cullin-1/5) assembled into a multi-subunit RING E3 ubiquitin ligase complex are substrates for neddylation that regulates the ubiquitination pathway important in NF-κB activity and pro-inflammatory cytokine production. In this study, we showed that upon live *Eh* contact with human macrophages, cullin-1/4A/4B/5 but not cullin-2/3, were degraded within 10 minutes. Similar degradation of cullin-1/5 were observed from colonic epithelial cells and proximal colonic loops tissues of mice inoculated with live *Eh*. Degradation of cullin-1/5 was dependent on *Eh*-induced activation of caspase-1 via the NLRP3 inflammasome. Unlike cullin-4B, the degradation of cullin-4A was partially dependent on caspase-1 and was inhibited with a pan caspase inhibitor. Cullin-1/5 degradation was dependent on *Eh* cysteine proteinases *Eh*CP-A1 and *Eh*CP-A4, but not *Eh*CP-A5, based on pharmacological inhibition of the cysteine proteinases and *Eh*CP-A5 deficient parasites. siRNA silencing of *cullin-1*/5 decreased the phosphorylation of pIκ-Bα in response to *Eh* and LPS stimulation and downregulated NF-κB-dependent TNF-α mRNA expression and TNF-α and MCP-1 pro-inflammatory cytokine production. These results unravel a unique outside-in strategy employed by *Eh* to attenuate NF-κB-dependent pro-inflammatory responses via NLRP3 activation of caspase-1 that degraded cullin-1/5 from macrophages.

## Author summary

The protozoan parasite *Entamoeba histolytica* (*Eh*) is the etiologic agent for the disease amebiasis. It is a potent pathogen that deploys an arsenal of virulence factors to trigger and subvert host immune defenses. One of the hallmark features of the disease is amebic colitis and in extreme cases, it can lead to abscesses of the liver and brain. For unknown reasons, the parasite breaches colonic mucosal barriers and invade underlying tissues. The host immune system plays a decisive role in determining the outcome of the disease. At the molecular level, the interaction of *Eh* with macrophage is a turning point in shaping pro-inflammatory responses. Understanding host-pathogen intricacies at the molecular level is key in determining the complexity of the disease. In the context of amebiasis, the underlying molecular events that occur at the *Eh*-macrophage intercellular junction are partly unravelled. Here we sought to interrogate the mechanisms by which NF-κB signaling is aborted following *Eh*-macrophage contact and found two regulatory scaffold proteins, cullin-1 and -5 (cullin-1/5) of the multiple E3 ligase complex, are degraded leading to dampening of NF-κB signaling. During *Eh*-macrophage contact, cullin-1/4A/4B/5 were rapidly degraded whereas cullin-2/3 were not. The degradation of cullin-1/5 was highly dependent on *Eh*-induced caspase-1 activation via the NLRP3 inflammasome. In contrast, the degradation of cullin-4A but not cullin-4B, was partially dependent on caspase-1 and was inhibited with a cell-permeable pan caspase inhibitor. Intriguingly, we found that *Eh* virulence factor *Eh*CP-A1 and *Eh*CP-A4, but not *Eh*CP-A5, played an important role in mediating the degradation of these proteins. Silencing cullin-1/5 decreased the phosphorylation of Iκ-Bα in response to *Eh* and LPS stimulation that markedly downregulated NF-κB-dependent TNF-α mRNA expression and TNF-α and MCP-1 pro-inflammatory cytokine production. This study unravelled a novel role for *Eh*-induced NLRP3 inflammasome activation of caspase-1 that intersected with the NF-κB pathway leading to the degradation of the novel substrates cullin-1/5 that regulates NF-κB-dependent pro-inflammatory cytokine production.

## Introduction

Amebiasis is caused by the extracellular enteric protozoan parasite *Entamoeba histolytica* (*Eh*), one of the leading causes of morbidity related to dysentery worldwide. *Eh* infects ~10% of the world population leading to 100,000 deaths/year from amebic colitis and liver abscess [1]. The disease is one of the leading causes of severe diarrhea in developing countries attributing to poor sanitation and nutrition [2]. The efficacy and quality of the host immune response determines the outcome of disease. For unresolved reasons, ~10% of *Eh* infection sporadically breaches innate mucosal barriers and invade the lamina propria. The root cause behind the development of symptomatic infection is not fully understood, but in part, is contributed by the quality of the immune response, and the expression of *Eh* virulence factors [3–5]. Invasion of the colonic mucosa by *Eh* leads to a robust pro-inflammatory cytokine burst entailing recruitment of immune cells including neutrophils and macrophages to the site of the infection [6–8]. *Eh* deploy an arsenal of virulence factors and the major ones include the galactose/N-acetyl-D-galactosamine (Gal/GalNAc) lectin (Gal-lectin), amoebapore, cysteine proteinases and prostaglandin E_2_ [5,9]. The Gal-lectin is a major surface component of *Eh* that mediates binding to host cells and Gal and GalNAc colonic MUC2 mucin glycans [10,11]. Among the different cysteine proteinases, *Eh*CP-A1, *Eh*CP-A2 and *Eh*CP-A5 are highly expressed in axenically cultured *Eh* [12,13]. The repertoire of cysteine proteinases is express spatially: *Eh*CP-A1 is confine to intracellular vesicles while *Eh*CP-A5 is expressed on the cell surface, and *Eh*CP-A2 is limited to the inner and outer cell membrane [14–16]. These cysteine proteinases have been one of the contributing factors in the pathogenesis of amebiasis [14,17,18].

In innate immunity, macrophages are instrumental in mounting a robust pro-inflammatory response when *Eh* invades the lamina propria to recruit additional immune cells to combat infection [19,20]. Elevated levels of TNF-α produced from macrophage has detrimental outcome leading to increased diarrheal disease during a primary infection. In host defense however, TNF-α and interferon-γ (IFN-γ) activated macrophages produce nitric oxide that kills *Eh* [20–22]. *Eh* Gal-lectin macrophage interaction modulates PAMPs (pathogen associated molecular patterns) via Toll-like receptors (TLR2 and TLR4) to trigger nuclear factor-κB (NF-κB) signaling leading to a pro-inflammatory burst of cytokines [9,23]. However, an unresolved issue is how does *Eh* dampens NF-κB mediated pro-inflammatory responses in macrophages? Apart from the Gal-lectin and *Eh*CP-A5 RGD ligation to α_5_β_1_ integrin on macrophages that activates caspase-1 via the NLRP3 inflammasome [24], we recently uncovered a role for *Eh*CP-A1 and *Eh*CP-A4 in the activation of caspase-6-dependent degradation of the cytoskeletal proteins (talin, Pyk2 and paxillin) that regulated IL-1β processing and secretion [25]. Curiously, in this interaction, NF-κB-dependent pro-inflammatory cytokine production (TNF-α and MCP-1) was negligible. These studies underscore that *Eh*-macrophage contact can activate multiple inflammatory caspases that can intersect several intracellular targets to enhance and/or regulate pro-inflammatory responses.

**Fig 1:**
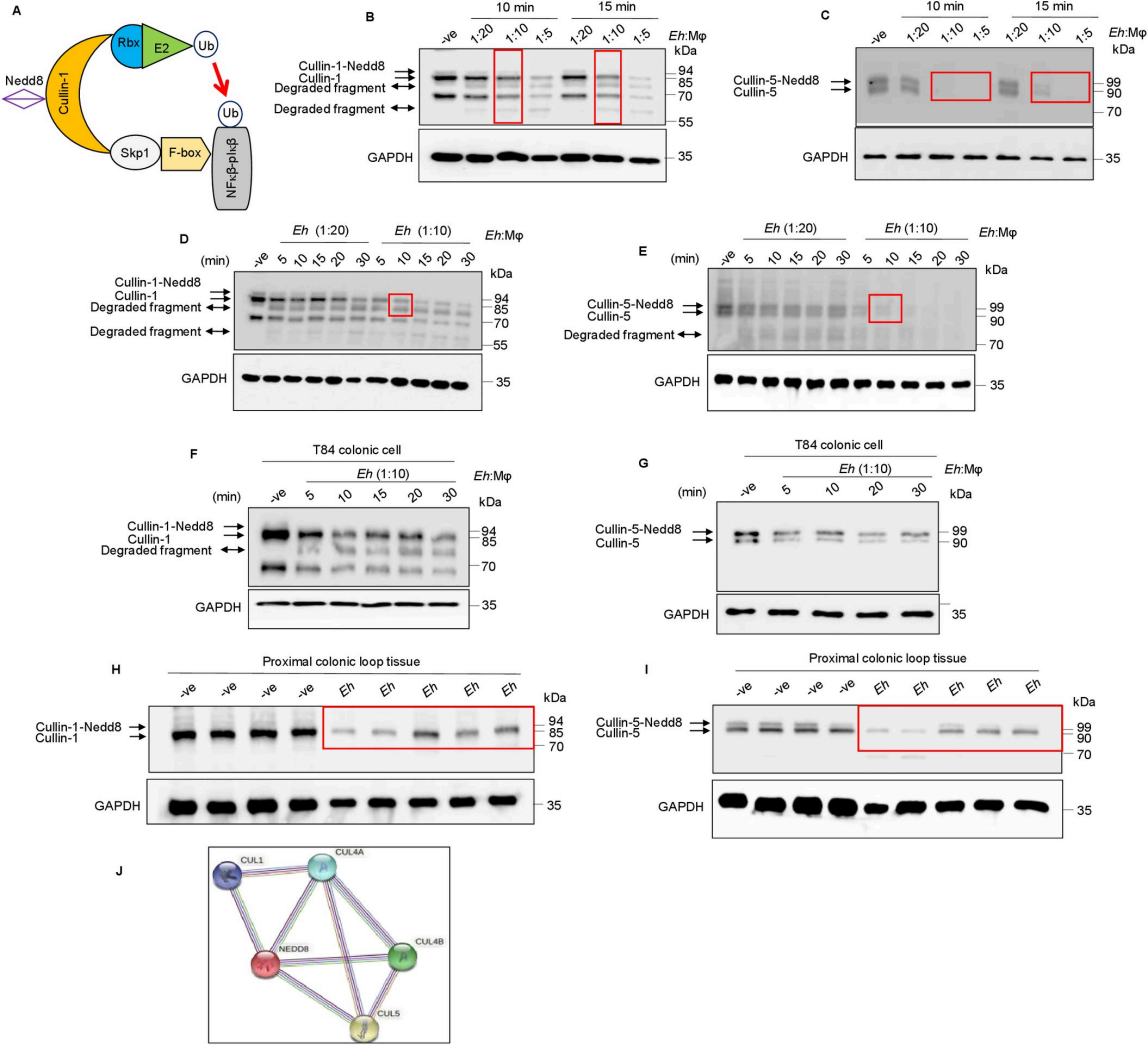
*Entamoeba histolytica* promotes the degradation of cullin-1/5 from THP-1 derived macrophages, colonic epithelial cells, and proximal colonic loop tissues in a time- and dose-dependent fashion. **(A)** Schematic representation of Skp-1-Cullin-1-F-box (SCF) protein E3 complex (Skp-1: S-phase kinase-associated protein 1; Rbx: RING [Really Interesting New Gene]-box1; E2: Ubiquitin-loaded E2). **(B, C)** THP-1 macrophages were incubated with different *Eh*:macrophage ratios, or **(D, E)** for increasing times (5 to 30 min) with *Eh* (20:1 ratio) and *Eh* (10:1 ratio). **(F, G)** T84 colonic epithelial cells were grown in 12-well plates and stimulated with *Eh* (10:1 ratio) for 5 to 30 min. **(H, I)** Proximal colonic loops were inoculated with *Eh* and cell lysates were prepared. Post *Eh* stimulations, cells were washed and cytoplasmic extracts were prepared, and an equal amount of protein was loaded on to the SDS- PAGE gel (7.5%) and immunoblotted with the anti-cullin-1, anti-cullin-5 and anti-GAPDH antibody. Highlighted boxed areas on the figures show point of interest for cullin-1/5 as described in text. **(J)** Protein-protein interaction using STRING v11 showed direct interaction between cullin-1, cullin-5, cullin-4A, cullin-4B and Nedd8. Data **(B, C, D, E, H, and I)** are representative of three independent experiments and data **(F and G)** are representative of two independent experiment and statistical significance was carried out with Students t- test. Bar represent mean ± SEM. **P<0.01, ***P<0.001.

Cullin proteins are molecular scaffolds that play an indispensable role in regulating post-translational modification of target proteins involving ubiquitin [26]. A diverse array of eukaryotic functions are controlled by the superfamily of ubiquitin ligase, known as the cullin-RING ligase (CRL) [27]. Cullin-1 is an extensively studied substrate of this pathway containing multiple Skp1-Cullin-1-F-box protein E3 complex that mediates the ubiquitination of its substrate, NF-κB (see **Fig 1A**) involved in cellular homeostasis [28]. This complex interacts with RING-domain protein Rbx-1 or Rbx-2 which engage charged ubiquitin E2 ligases into the complex leading to the ubiquitination of its substrate, cullin [29]. Mammalian cells express different cullin proteins (cullin-1, cullin-2, cullin-3, cullin-4A, cullin-4B and cullin-5) which are modified post-translationally by Nedd8 [30,31]. Among these, cullin-5 was shown to be involved in regulating the ubiquitination of TRAF-6 (TNF receptor-associated factor 6) thus, modulating signaling via LPS [32].

The aim of this study was to determine the molecular mechanisms that regulates NF-κB-dependent pro-inflammatory responses following *Eh*-macrophage contact. We have uncovered that following *Eh*-macrophage contact mediated by the Gal-lectin and *Eh*CP-A1 and *Eh*CP-A4, but not *Eh*CP-A5, caspase-1 was activated via the NLRP3 inflammasome pathway that degraded the substrates cullin-1/5 to inhibit NF-κB signaling. Silencing cullin-1/5 decreased the phosphorylation of pIκ-Bα in response to *Eh* and LPS stimulation and downregulated NF-κB-dependent TNF-α mRNA expression and production of the pro-inflammatory cytokines, TNF-α and MCP-1. These results unravel a unique outside-in signaling upon *Eh*-macrophage contact that attenuated NF-κB-dependent pro-inflammatory cytokine responses by caspase-1 that mediated the degradation of cullin-1/5.

## Results

### *E*. *histolytica* (*Eh*) promotes the degradation of cullin-1 and cullin-5 from macrophages and colonic epithelial cells in a dose- and time-dependent fashion

*Eh* is known to modulate cell signaling pathways by promoting the degradation of proteins from host cells [33,34]. We have recently shown that upon *Eh*-macrophage contact, caspase-6 was activated that cleaved the cytoskeletal proteins talin, Pyk2 and paxillin that caused robust secretion of the pro-inflammatory cytokine IL-1β whereas, NF-κB-dependent pro-inflammatory cytokine production was unaffected [25]. This unique pro-inflammatory signature is dependent upon live *Eh* in direct contact with macrophages that activates the NLRP3 inflammasome pathway [24,35–37], as purified native Gal-lectin is a potent stimulus for TNF-α transcription and protein release [20,24,38–40]. To address mechanistically why *Eh* in contact with macrophages attenuates NF-κB signaling, we hypothesized that *Eh*-induced inflammatory caspases could be destabilizing the NF-κB complex. A likely candidate is cullin-1/5 scaffolding proteins of the E3 ligase complex that play a crucial role in maintaining normal cellular homeostasis by regulating the ubiquitination of several proteins including, NF-κB (**Fig 1A**). Neddylation is a post-translational modification that regulates the activity of cullin-1/5. In resting human macrophages, cullin-1/5 appears as a doublet (**Fig 1B and 1C)**. For cullin-1, the lower band (~85 kDa) is unneddylated while the upper band (~94 kDa) is neddylated (Cullin-1-Nedd8; **Fig 1B**). A prominent band (~68 kda) was detected using a cullin-1 antibody obtained from Thermo Fisher that could be an isoform or a truncated form of cullin-1. This protein was not observed using a different antibody from Cell Signalling in the dose-dependent experiments (**S1A Fig**). As the Thermo Fisher antibody showed high immunoreactivity in detecting the degraded fragments it was used for all subsequent studies. For cullin-5, the lower band (~90 kDa) is unneddylated while the upper band (~99 kDa) is neddylated (Cullin-5-Nedd8; **Fig 1C**). To distinguish between the two bands, macrophages were incubated with the known neddylation inhibitor, MLN4924 that inhibited the upper bands of cullin-1/5 with no change in the lower band corresponding to uneddylated cullin (**S1B and S1C Fig**). Global neddylation profiling during *Eh*-macrophage (1:10) contact as early as 10 min, showed a marked increase in total neddylated proteins with a concomitant decrease in free monomeric Nedd8 (**S1D Fig**). We also investigated if the other cullins were degraded from macrophages and found that similar to cullin-1/5 (**S2A and S2B Fig**), cullin-4A/B (**S2C and S2D Fig)** were rapidly degraded within 10 min, while cullin-2/3 (**S2E and S2F Fig**) were not.

*Eh* in contact with macrophages significantly degraded both neddylated (Nedd8) and unneddylated cullin-1/5 in a dose-dependent fashion (**Figs 1B and 1C and S3A and S3B**). Degradation of cullin-1/5 started as early as 10 min **(Fig 1D and 1E)** with complete disappearance of neddylated cullin concomitant with a decrease in unneddylated cullin and the appearance of a two cleavage fragments at ~62 and ~81kDa for cullin-1 (**Fig 1D**) and degraded fragments at ~80 kDa for cullin-5 (**Fig 1E**). Sub-optimal degradation of cullin-1/5 occurred after 10 min with 1:10 *Eh* to macrophage ratio (**Figs 1D and 1E and S3C and S3D**) and this ratio was used for all subsequent studies. *Eh* did not increase the neddylation of cullin-1/5 (**Fig 1D and 1E**) above basal levels. Degradation of cullin-1/5 was not restricted to macrophages as *Eh* in contact with human T84 colonic epithelial cells showed similar degradation of cullin-1 (**Fig 1F**) and -5 (**Fig 1G**) in a time-dependent manner. To determine if cullin-1/5 was degraded during intestinal amebiasis, we used a short-term mouse colonic loop model of infection and showed significant degradation of neddylated and unneddylated cullin-1 (**Figs 1H and S3E**) and -5 (**Figs 1I and S3F**). A similar dose- and time-dependent degradation pattern was observed with *Eh* in contact with bone marrow-derived macrophages (BMDM) for cullin-1 (**S4A and S4B Fig**) and -5 (**S4C and S4D Fig**). Protein-protein interaction using STRING v11 (https://string-db.org) showed a synergistic liaison between cullin-1 (CUL1), cullin-4A (CUL4A), cullin-4B (CUL4B), cullin-5 (CUL5) and NEDD8 (**Fig 1J**) that may in part, explain the association of the protein complex susceptibility to degradation. Taken together, these results demonstrate for the first time that cullin-1/4A/4B/5 are novel substrates that are degraded upon *Eh*-macrophage contact.

### Cullin-1/5/4A/4B degradation is dependent on contact with live *E*. *histolytica*

**Fig 2:**
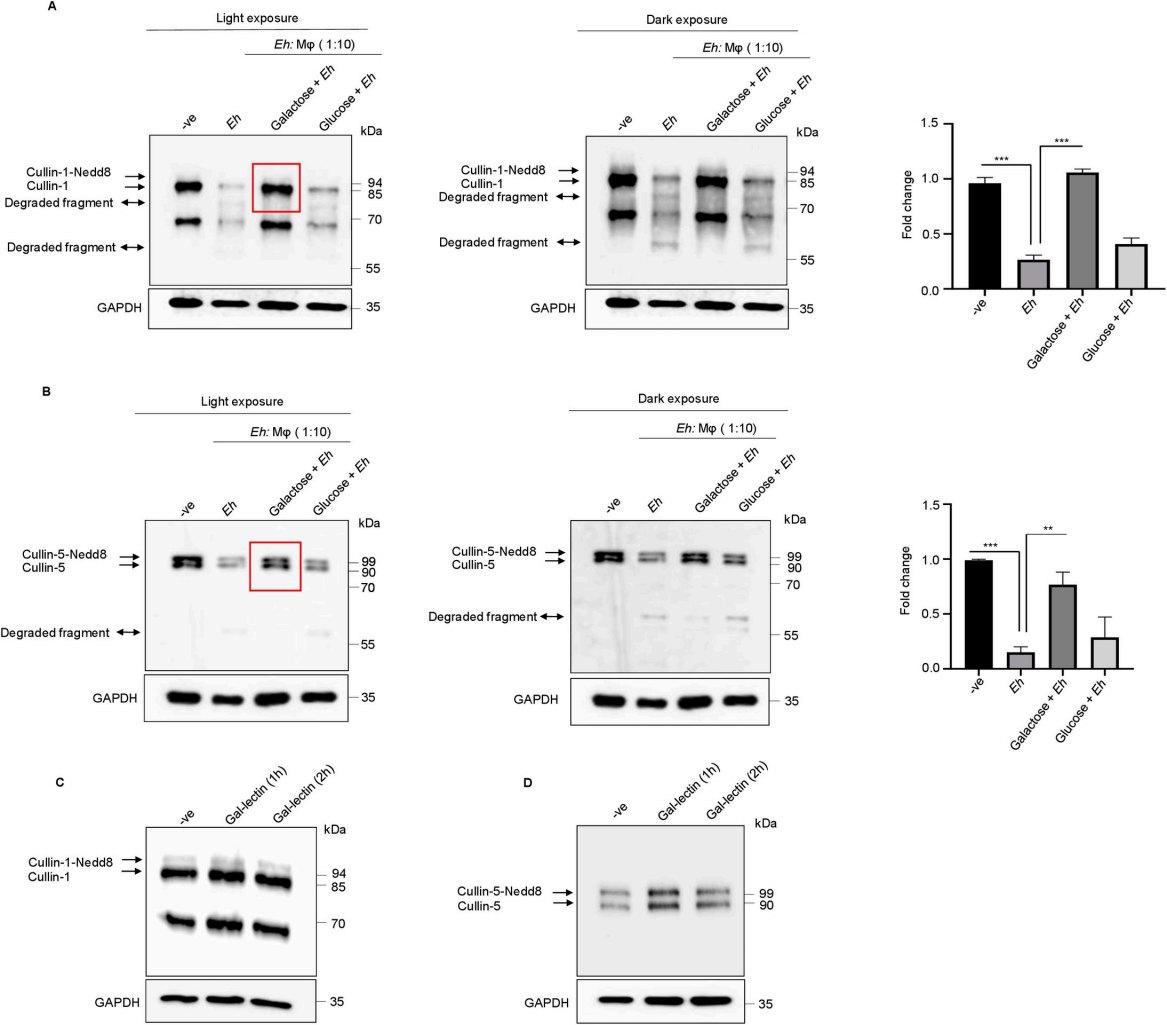
Cullin-1/5 degradation requires Gal-lectin-mediated contact with macrophage. **(A, B)** THP-1 macrophages were stimulated with either *Eh* (10:1 ratio) alone or with *Eh* preincubated with 55mM Galactose or Glucose for 10 min at room temperature and the degradation of the cullin-1/5 determined. **(C, D)** THP-1 macrophages were stimulated with native *Eh* Gal-lectin (500ng/ml) for 1h and 2h. Post incubation, cells were washed and lysed. Equal amount of protein was loaded onto 7.5% SDS-PAGE gel and immunoblotted with anti-cullin-1, anti-cullin-5 and anti-GAPDH antibody. Highlighted boxed areas on the figures show point of interest for cullin-1/5 as described in text. Data are representative of three independent experiments and statistical significance was carried out with Students t- test. Bar represent mean ± SEM. **P<0.01, ***P<0.001.

We next determined the requirement for live *Eh* in contact with macrophages responsible for triggering the degradation of cullin. *Eh* Gal lectin is the major surface adhesin molecule that mediates high affinity binding to target cells including macrophage [11,41]. We have previously shown [25] that live *Eh*, but not *Eh* soluble components or membranes in contact with macrophages activated caspase-6 that initiated the degradation of cytoskeletal proteins and speculated that a similar mechanism may occur in this study. Consistent with our previous studies, competitively blocking *Eh* Gal-lectin from binding macrophages with exogenous galactose, significantly inhibited the degradation of both forms (neddylated and unneddylated) of cullin-1 (**Fig 2A**) and -5 (**Fig 2B**) whereas, glucose as an osmotic control, had no inhibitory effects. Native purified Gal-lectin signals via the NF-κB pathway to regulate TNF-α [20,24,38–40] but loses this effect in live *Eh* in the presence of other adhesins (e.g., *Eh* cysteine proteinases). In support of this, stimulation of macrophages with purified native soluble Gal-lectin alone did not trigger the degradation of cullin-1 (**Fig 2C**) and -5 (**Fig 2D**), reinforcing the fact that live *Eh* engages multiple receptors on the surface of macrophage to activate inflammatory caspases and/or other pathways as compared to soluble *Eh* proteins alone. These results point towards an indispensable role for the Gal-lectin as a co-receptor (bridge) together with other *Eh* surface proteins to trigger the cleavage of cullin. To corroborate this hypothesis, we investigated whether fixed *Eh* and *Eh* lysate could trigger the cleavage of cullin-1/4A/4B/5 in macrophages. As predicted, only live *Eh* concomitant with the activation of caspase-1 (see section below on caspase-1), but not fixed *Eh* or *Eh* lysate degraded cullin-1/4A/4B/5 (**S5 Fig**). These results show a distinct requirement for live *Eh* in direct contact with macrophages to trigger the degradation cullin-1//4A/4B/5 (**S2A–S2D Fig**).

### Cullin-1 and -5 degradation is dependent on *Eh*CP-A1 and *Eh*CP-A4 but independent of *Eh*CP-A5

**Fig 3:**
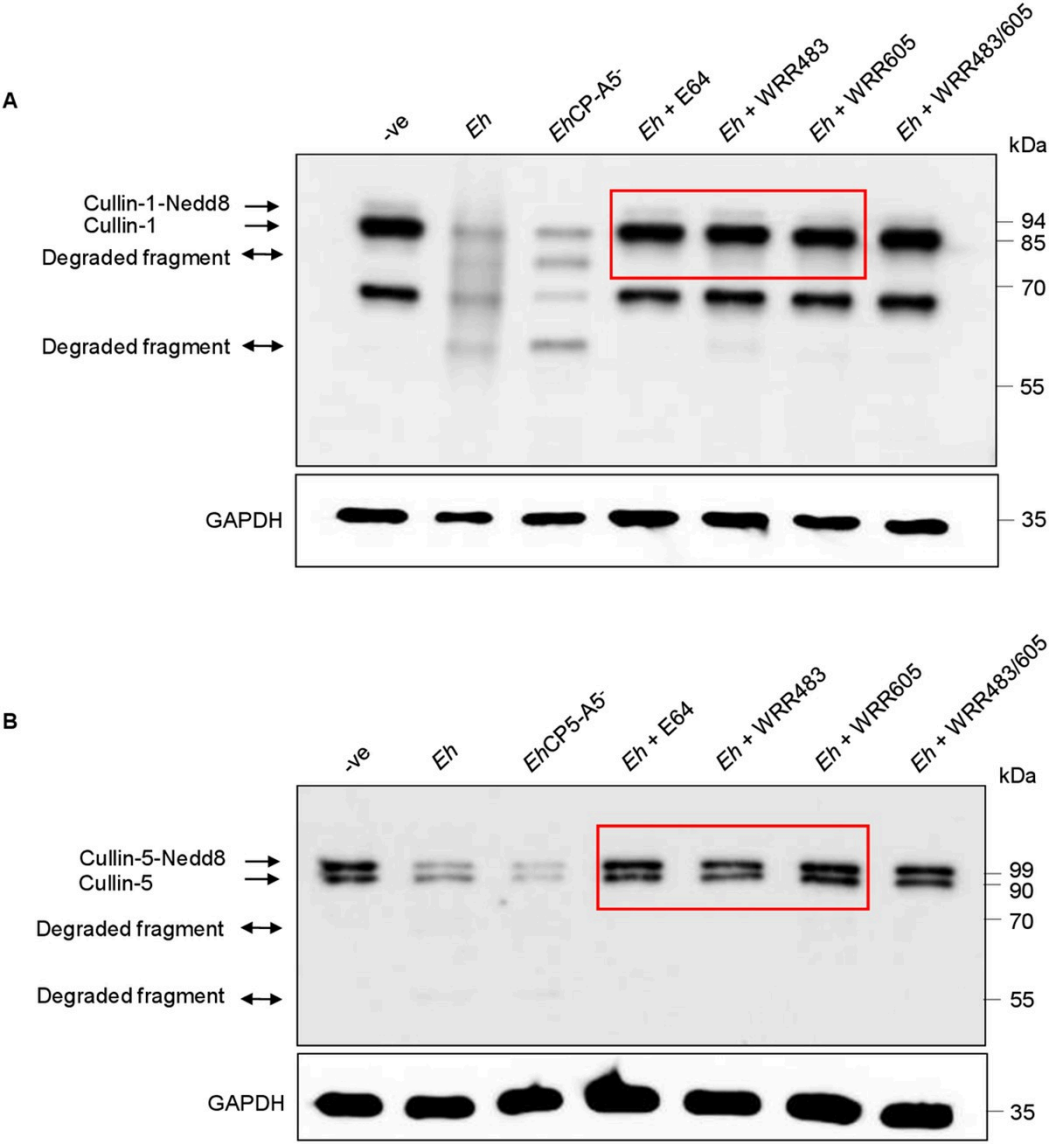
Cullin-1/5 degradation requires *Eh*CP-A1 and *Eh*CP-A4 but independent of *Eh*CP-A5. **(A, B)** THP-1 derived macrophages were incubated with wild type *Eh* (10:1 ratio), *Eh*CP-A5 deficient *Eh* (*Eh*CP-A5^-^), E64 inhibitor-treated *Eh*, *Eh* pre-incubated with WRR483, (inhibitor for *Eh*CP-A1), WRR 605 (inhibitor for *Eh*CP-A4) or *Eh* pre-treated with both WRR483 and WRR605 in combination for 30 min at 37° C for 10 min. Post incubation, cells were washed and lysed and equal amount of protein was loaded onto SDS- PAGE gel and immunoblotted with anti-cullin-1 **(A)**, anti-cullin-5 **(B)** and anti-GAPDH antibody. Highlighted boxed areas on the figures show point of interest for cullin-1/5 as described in text. Results are representative of two independent experiments.

*Eh* cysteine proteinases play an important role in imparting virulence and modulating host cell defense responses [42]. Depending the cell type, *Eh*CP-A5 RGD motif binds α_5_β_1_ integrin on macrophages to activate caspase-1 via the NLRP3 inflammasome [24] or α_v_β_3_ integrin on colonic cells to stimulate NF-κB pro-inflammatory responses [43] and MUC2 mucin exocytosis [44]. To assess if *Eh*CPs were playing a role in triggering the degradation of cullin-1/5, *Eh* was pretreated with E64, a broad-spectrum cysteine proteinases inhibitor and the degradation of cullin-1(**Fig 3A**) and -5 (**Fig 3B**) was rescued as compared to untreated *Eh*. To investigate which specific *Eh* cysteine proteinase was involved, macrophages were stimulated with *Eh*CP-A5 deficient *Eh* that degraded cullin-1 (**Fig 3A**) and -5 (**Fig 3B**) similar to wild type *Eh* indicating that other *Eh*CPs were involved as E64 treated *Eh* completely blocked the degradation of cullin-1/5. Likely candidates are *Eh*CP-A1 and *Eh*CP-A4 given their role in the activation of caspase-6 in contact with macrophages that initiated the cleavage of cytoskeletal proteins at the contact site [26]. To address this, macrophage was stimulated with *Eh* pre-treated with either WRR483 (*Eh*CP-A1 inhibitor) or WRR605 (*Eh*CP-A4 inhibitor) or in combination. Intriguingly, WRR483 and WRR605 completely rescued the cleavage of both neddylated and unneddylated cullin-1(**Fig 3A**) and -5 (**Fig 3B**). *Eh* pretreated with both enzyme inhibitors in combination (WRRR483 and WRR605) had a similar effect as E64 in rescuing the degradation of cullin-1/5 and was identical to the negative control. These results directly implicate the involvement of *Eh*CP-A1 and *Eh*CP-A4 in mediating the degradation of cullin-1/5 following Gal-lectin mediated contact with macrophage.

### Potential cleavage site prediction by caspases in cullin using the software, peptide cutter

**Fig 4:**
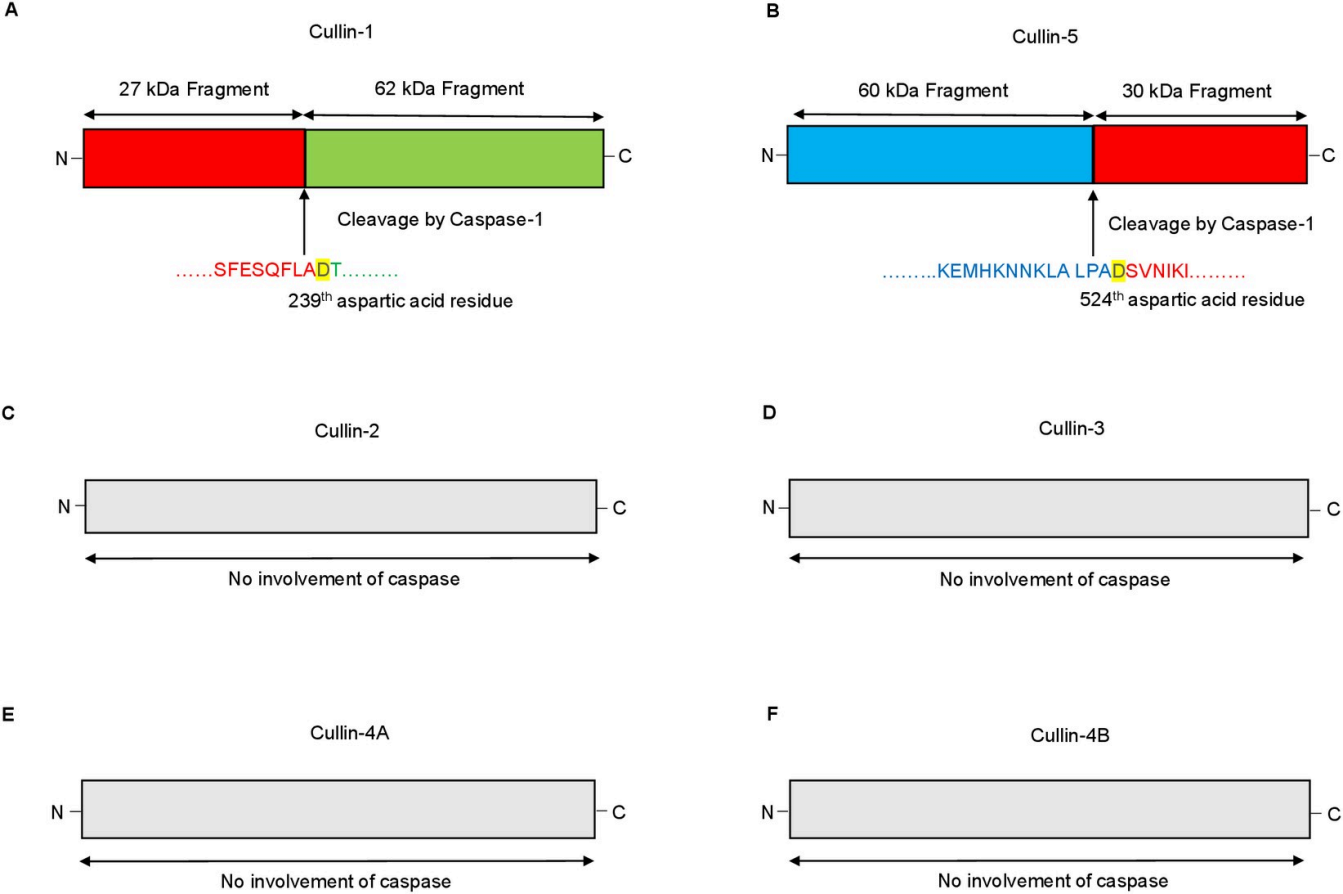
Potential cleavage site prediction by caspases in different cullin using the software peptide cutter. The software Peptide Cutter (ExPASy) that predicts potential cleavage sites by different chemicals and proteases against a given query (Protein sequence) was used, for determining the potential cleavage site in different cullin protein’s (cullin-1, cullin-2, cullin-3, cullin-4A, cullin-4B and cullin-5). **(A)** The yellow color at D^239^, aspartic acid residue (arrow) represents the potential site for cullin-1 cleavage by caspase-1. The sequence in red represents a fragment of 27 kDa and the sequence in green represents a fragment of 62 KDa. (**B)** The yellow color at D^524^, aspartic acid residue (arrow) denotes the potential cleavage site for cullin-5 by caspase-1. The sequence in blue represents a fragment of 60 kDa, while the sequence marked in red represents a 30 kDa fragment. **(C-F)** No potential cleavage sites for caspase-1 were noted for cullin-2, 3, 4A and 4B.

Recent advances in the interaction between *Eh* and the host has shown an intriguing relationship between different caspases [36]. Upon *Eh* contact with macrophages, activated caspase-1 and caspase-4 formed a protein complex that enhanced the cleavage of caspase-1 CARD domains to augment IL-1β release and cleavage of gasdermin D that regulated pro-inflammatory cytokine release [37]. *Eh* in contact with macrophages also activate caspase-6 that mediates cytoskeletal-associated protein degradation [25] and caspase-3 important for apoptosis [45]. To determine if inflammatory caspases played a role in cleaving cullin-1/5, we used the bioinformatic software Peptide Cutter (ExPASy) (https://web.expasy.org/peptide_cutter/) that predicts potential cleavage sites by different proteinases against a given protein sequence and found cullin-1 (**Fig 4A**) and cullin-5 (**Fig 4B**) are targets for caspase-1 cleavage. The software predicted the involvement of D^239^ in cullin-1 (**Fig 4A**) to be a potential cleavage site for caspase-1 yielding two different fragments of 27 and 62 kDa, respectively. The cleavage site for cullin-5 (**Fig 4B**) by caspase-1, is D^524^ residue resulting in two different fragments of 30 and 60 kDa. There were no putative caspase-1 cleavage sites in the other cullin proteins: cullin-2 **(Fig 4C)**, cullin-3 **(Fig 4D)**, cullin-4A **(Fig 4E)** and cullin-4B **(Fig 4F)**. These findings point towards a definite role for caspase-1 in mediating the cleavage of cullin-1/5.

### Cullin-1/4A/5 degradation is mediated by caspase-1 during *E*. *histolytica* contact with macrophage

**Fig 5:**
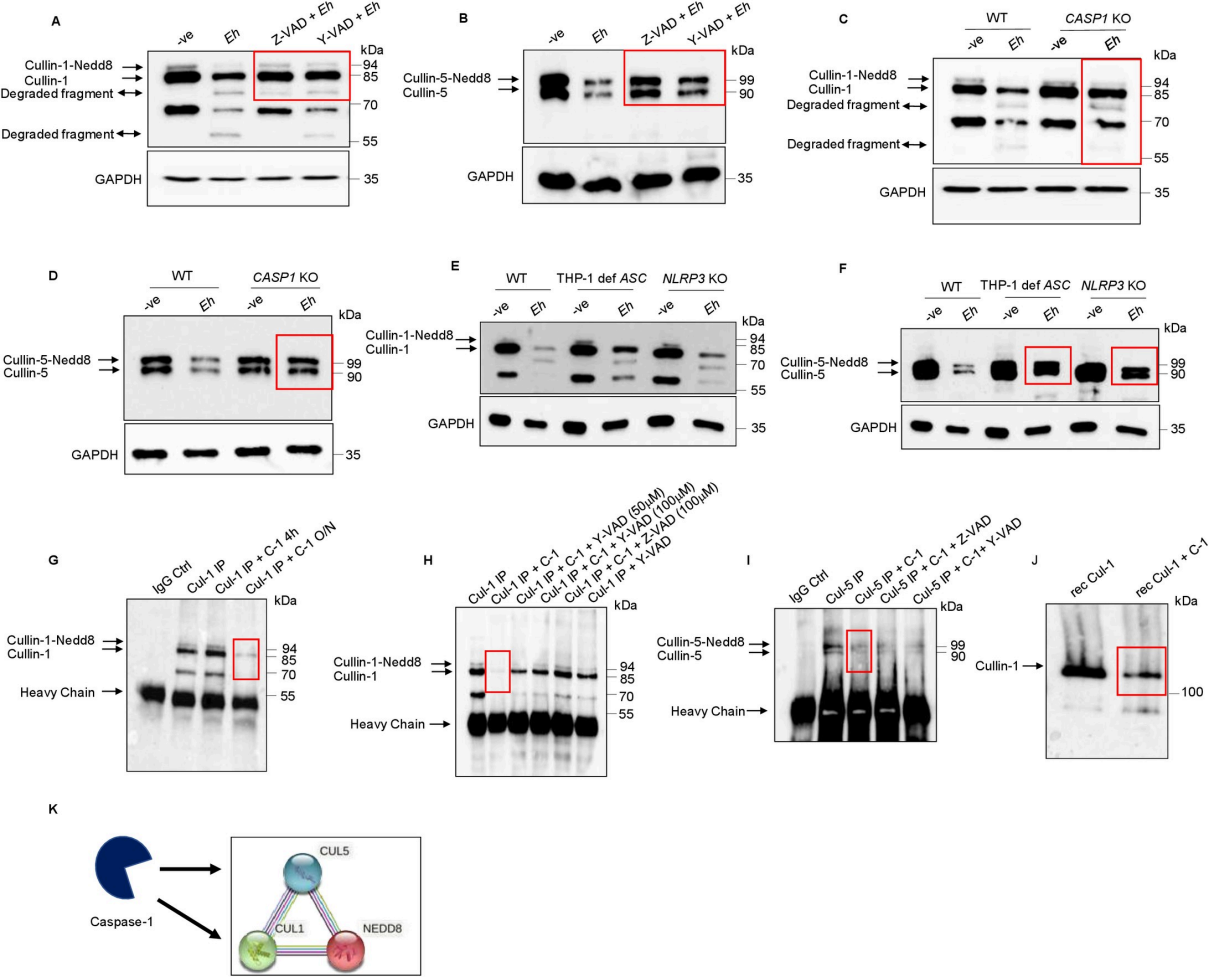
Caspase-1 activation during *Eh-*macrophage contact degrades cullin-1/5. (**A, B)** THP-1 derived macrophages were pre-incubated with the pan-caspase inhibitor Z-VAD-fmk (100μM) and caspase-1 specific inhibitor Z-YVAD-fmk (100μM) for 1 h followed by stimulation with *Eh* (10:1 ratio) for 10 min. Cleavage of the cullin-1/5 were assessed by western blot. **(C, D)** Wild type (WT) THP-1 and *CASP1* CRISPR/Cas9-KO macrophages were stimulated with *Eh* (10:1 ratio) for 10 min. **(E, F)** WT, THP-1 def *ASC* and *NLRP3* CRISPR/Cas9-KO THP-1 macrophages were stimulated with *Eh* (10:1 ratio) for 10 min. After incubation, cells were washed and lysed. Equal amount of protein was loaded on to the SDS-PAGE (7.5%) and immunoblotted with anti-cullin-1 **(**Panel: A, C, and E**)**, anti-cullin-5 (Panel: B, D and F) and anti-GAPDH antibody. **(G)** Cullin-1 was immunoprecipitated (Cul-1 IP) using anti cullin-1 antibody and post immunoprecipitation it was incubated with recombinant caspase-1 (C-1) for 4h or overnight (O/N). **(H)** Immunoprecipitated cullin-1 was incubated with recombinant caspase-1 or with Y-VAD-fmk (50μM or 100μM) or Z-VAD-fmk (100μM). **(I)** Immunoprecipitated cullin-5 was incubated with recombinant caspase-1 or with Z-VAD-fmk (100μM) or Y-VAD-fmk (100μM). **(J)** Recombinant cullin-1 (rec Cul-1) was incubated with recombinant caspase-1 overnight at 37° and was immunoblotted with anti-cullin-1 antibody. Note, recombinant cullin-1 (rec Cul-1) has a molecular weight of ~118 kDa due to the N-terminus GST tag (~28kDa). The highlighted boxed areas in the figures show point of interest for cullin-1/5 as described in text. **(K)** Protein-protein interaction using STRING v11 showed direct interaction between cullin-1, cullin-5 and Nedd8 demonstrated that cullin-1/5 are novel substrates for caspase-1. Data **(A, B, C and D)** are representative of three independent experiments while data **(E, F, G, I, and J)** are representative of two independent experiments.

*Eh* activates different inflammatory caspases upon contact with host cells [46]. Based on the bioinformatic findings above, we next investigated if caspase-1 could degrade the cullins. To investigate this, macrophages were pre-incubated with the pan-caspase inhibitor, Z-VAD-fmk, followed by *Eh* stimulation and it significantly inhibited the degradation of cullin-1 (**Figs 5A and S6A**), and -5 (**Figs 5B and S6B**) as compared to untreated cells stimulated with *Eh*, potentially implicating a role for caspases in mediating the degradation of cullin-1/5. To investigate specificity for caspase-1, macrophages were pre-incubated with the known caspase-1 inhibitor, Y-VAD-fmk, following by *Eh* stimulation, that rescued the degradation of cullin-1 (**Figs 5A and S6A**) and -5 (**Figs 5B and S6B**) as compared to *Eh* stimulated cells and the negative control. Specificity for caspase-1 was confirmed in *CASP1* CRISPR/Cas9 KO THP-1 macrophage that completely inhibited *Eh*-induced degradation of cullin-1 (**Figs 5C and S6C**) and -5 (**Figs 5D and S6D**). Surprisingly, the degradation of cullin-4A was modestly restored from Z-VAD-fmk and Y-VAD-fmk pre-incubated macrophages as well as from *CASP1* CRISPR/Cas9 KO THP-1 cell following *Eh* stimulation (**S6E Fig**) indicating partial involvement of caspase-1 in mediating the degradation of cullin-4A. In contrast, the degradation of cullin-4B was only weakly restored by Z-VAD-fmk but not with Y-VAD-fmk or in *CASP1* CRISPR/Cas9 KO THP-1 macrophage following *Eh* stimulation, which rules out the involvement of caspase-1 (**S6F Fig**).

To determine a role for *Eh*-induced NLRP3 inflammasome activation of caspase-1, WT THP-1, THP-1 def *ASC* and *NLRP3* CRISPR/Cas9 KO macrophages were stimulated with *Eh* and predictably, in THP-1 def *ASC* and *NLRP3* CRISPR/Cas9 KO macrophages, unneddylated forms of cullin-1 (**Fig 5E**) and both forms of cullin-5 (**Fig 5F**) were partially rescued from degradation. To quantify the kinetics of caspase-1 activation with concomitant degradation of cullin-1/5, macrophages were stimulated with *Eh* from 5 to 30 min in the presence/absence of caspase-1 inhibitors and LPS + Nigericin as a positive control for caspase-1 activation. *Eh*-induced the activation of caspase-1 CARD domain as early as 10 min (faint band at 10 min) with simultaneous degradation of cullin-1/5 that was completely rescued with Z-VAD and Y-VAD **(S7A Fig)**. Interestingly, macrophages stimulated with LPS + Nigericin activated caspase-1 that also degraded cullin-1/5 (**S7A Fig**) suggesting that other stimulus of caspase-1 can induce the degradation of cullin-1/5.

To determine substrate specificity of cullin-1/5 for caspase-1, we next investigated whether recombinant caspase-1 could directly cleave immunoprecipitated cullin-1/5 from THP-1 macrophages. As predicted, recombinant caspase-1 cleaved both forms of cullin-1 (**Fig 5G and 5H**) and -5 (**Fig 5I**) that was inhibited with Y-VAD and Z-YVAD-fmk (**Fig 5H and 5I**). To validate cullin degradation, the immunoprecipitated cullin-1 complex was incubated with recombinant caspase-1 overnight and by silver stain showed degradation both forms of cullin-1 (**S7B Fig**). Recombinant caspase-1 was also very efficient in cleaving recombinant cullin-1 (**Fig 5J**). Protein-protein interaction using the software STRING v11 showed strong interaction of cullin-1/5 to NEDD8, demonstrating for the first time that these proteins are novel targets for caspase-1 (**Fig 5K**). These findings clearly show that cullin-1/5 are targets for degradation by caspase-1 following *Eh*-macrophage contact.

### Cullin-1 and -5 are required for NF-κB signaling in response to *E*. *histolytica*

**Fig 6:**
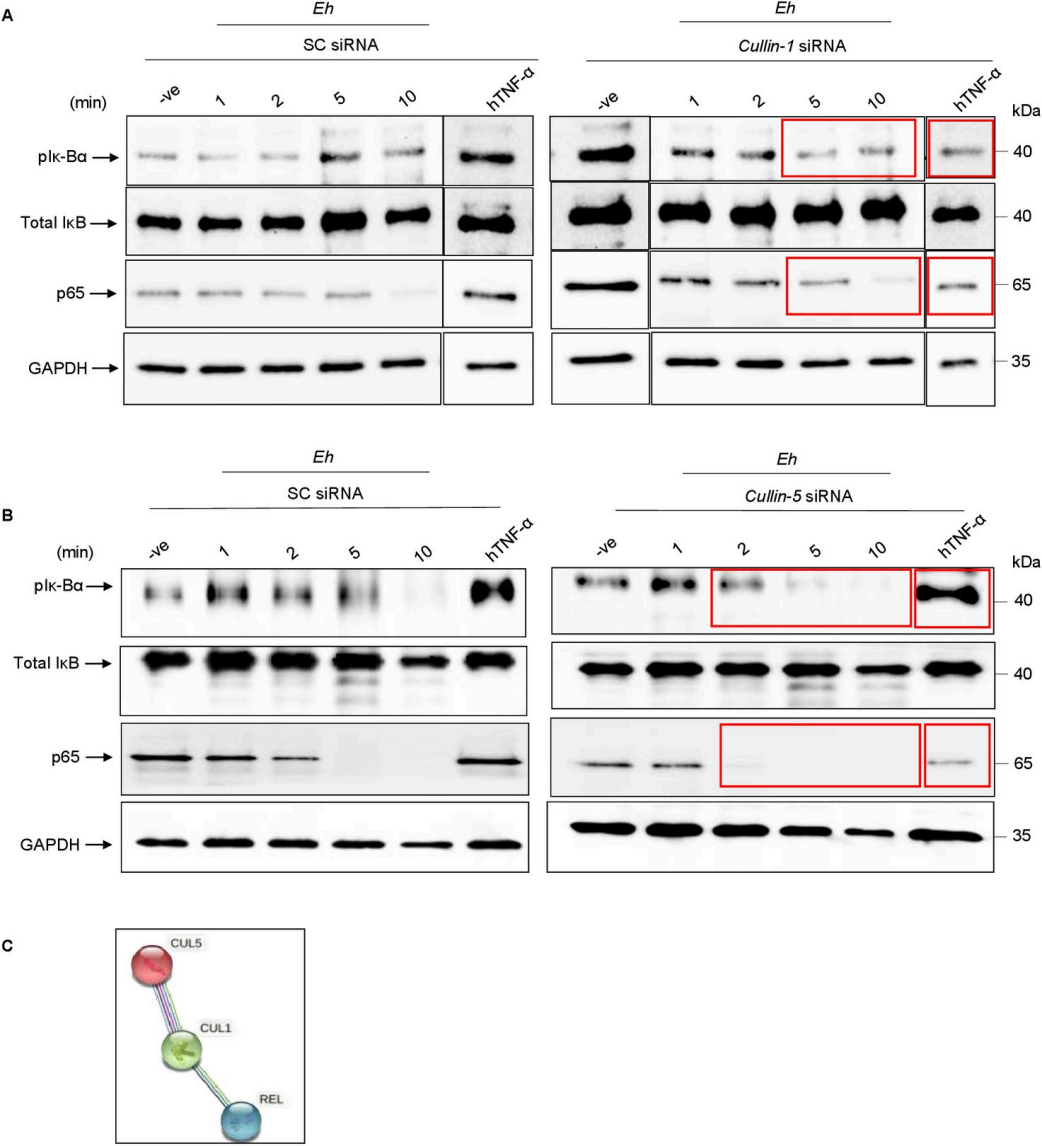
Cullin-1/5 are required for NF-κB signaling during *Eh*-macrophage contact. **(A)** THP-1 cells were transfected with 100nM of cullin-1 siRNA and scrambled control (SC siRNA) and in **(B)** 100nM of cullin-5 siRNA and scrambled control (SC siRNA). After 72h, cells were stimulated with *Eh* for 1-, 2-, 5- and 10-min. Recombinant human TNF-α (hTNF-α) was used as a positive control for the experiment. Post incubation with *Eh*, cells were washed and lysed, and an equal amount of lysate was loaded onto the SDS-PAGE gel (12%) and immunoblotted with anti-pIκ-Bα, anti-Iκ-B, anti-p65 and anti-GAPDH antibody. Highlighted boxed areas on the figures show decrease in pIκ-Bα and p65. **(C)** Protein-protein interaction using STRING v11 showed direct interactions between cullin-1 and Rel and direct interaction between cullin-1/5. Data are representative of two independent experiment.

We next investigated the role of cullin-1/5 in modulating NF-κB signaling in response to *Eh*. NF-κB exists as a heterodimer bound to inhibitory κB (IκB) proteins in the cytosol. Activation of the NF-κB pathway is dependent on the phosphorylation of Iκ-Bα ensuing the release of NF-κB dimers [47]. Since NF-κB dimer is an important target for cullin-1[48], we next sought to investigate if cullin-1/5 degradation affected the phosphorylation of Iκ-Bα, a marker for NF-κB activation. To explore this, cullin-1/5 was silenced by siRNA followed by *Eh* stimulation for different times. Silencing *cullin-1* decreased the levels of the pIκ-Bα as early as 5 min and for *cullin-5* as early as 2 min (**Fig 6A and 6B**). In *cullin-1/5* silenced cells, the expression of p65 was markedly decreased (**Fig 6A and 6B**). Recombinant hTNF-α was used as a positive control for NF-κB signaling that enhanced the levels of pIκ-Bα and p65 from scramble siRNA cells but not in *cullin-1/5* silenced cells (**Fig 6A and 6B**), directly implicating the involvement of cullin-1/5 in modulating NF-κB signaling. Rel/NF-κB transcription factors are regulators of inflammatory acute phase responses and play an indispensable role in apoptosis and cell proliferation [49]. Protein-protein interaction using STRING v11 showed a direct interaction between cullin-1 (CUL1) and NF-κB (REL) while it was indirectly interacting with cullin-5 (CUL5, **Fig 6C**). These results show an interesting mechanism initiated by *Eh* to decrease the levels of pIκ-Bα by degrading both cullin-1/5 to abort NF-κB signaling.

### Silencing of cullin-1 and -5 attenuated the expression of NF-κB dependent cytokines

**Fig 7:**
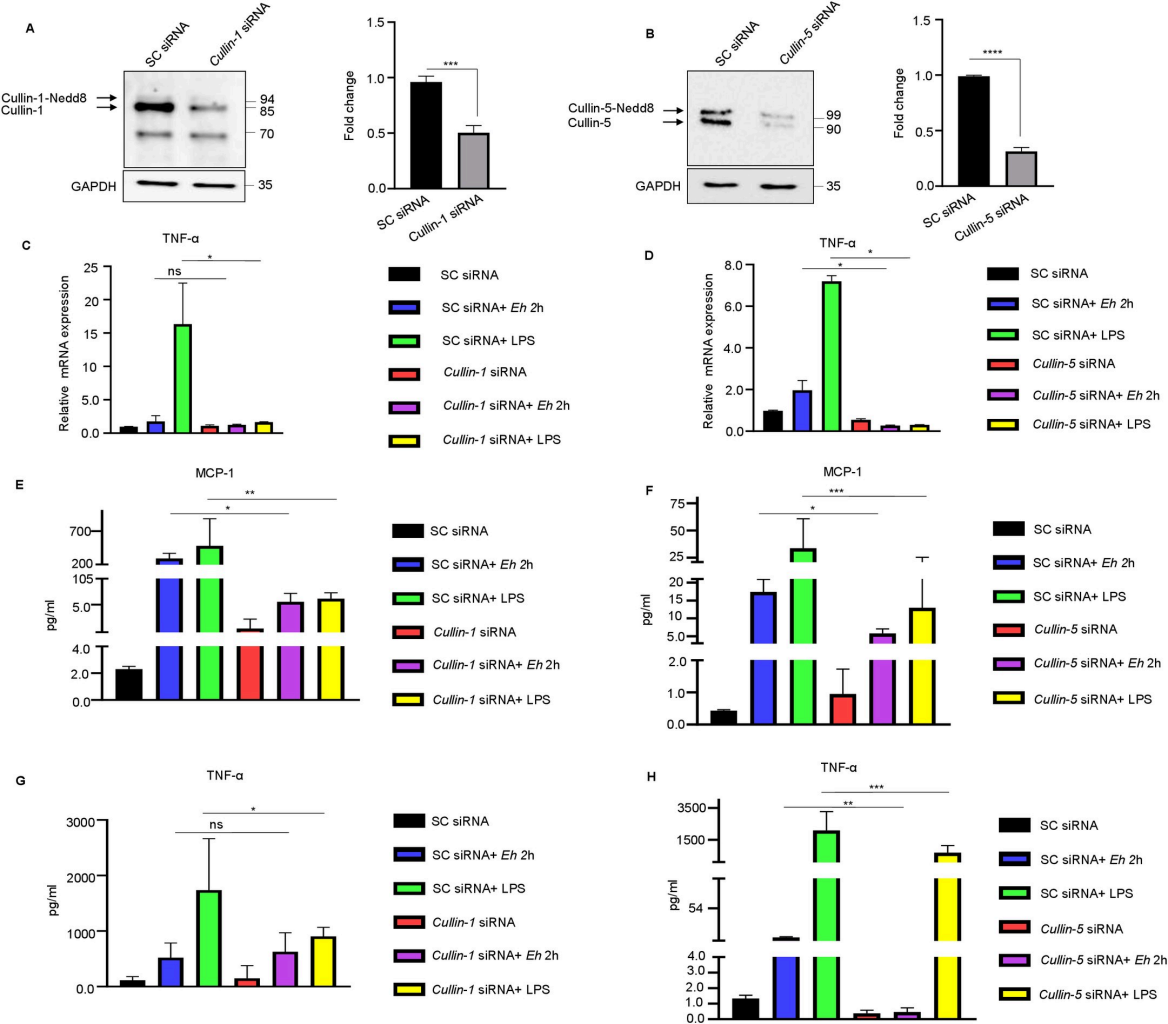
Silencing *cullin-1/5* decreased mRNA and protein expression of NF-κB dependent cytokines. **(A, B)** THP-1 cells were transfected with scrambled control (SC siRNA), 100nM of cullin-1 siRNA **(A)**, and 100nM of cullin-5 siRNA **(B)**, respectively. After 72h, transfected cells were washed and lysed and immunoblotted against the indicated antibody to verify silencing. (**C-D)** THP-1 cells silenced for cullin-1/5 by siRNA were stimulated with *Eh* for 2h and TNF-α mRNA expression was measured using real-time PCR. Post silencing of cullin-1/5 genes, cells were incubated with *Eh* for 2h and pro-inflammatory cytokines levels measured using human cytokine array pro-inflammatory focused 15-plex discovery assay. LPS was used as a positive control for the experiment. Silencing of cullin-1/5 downregulated MCP-1 **(E, F)** and TNF-α (**G, H)** in response to LPS stimulation. Data are representative of three independent experiments. Bars represent mean ± SEM. * P <0.05, ** P <0.01, ***P <0.001.

**Fig 8:**
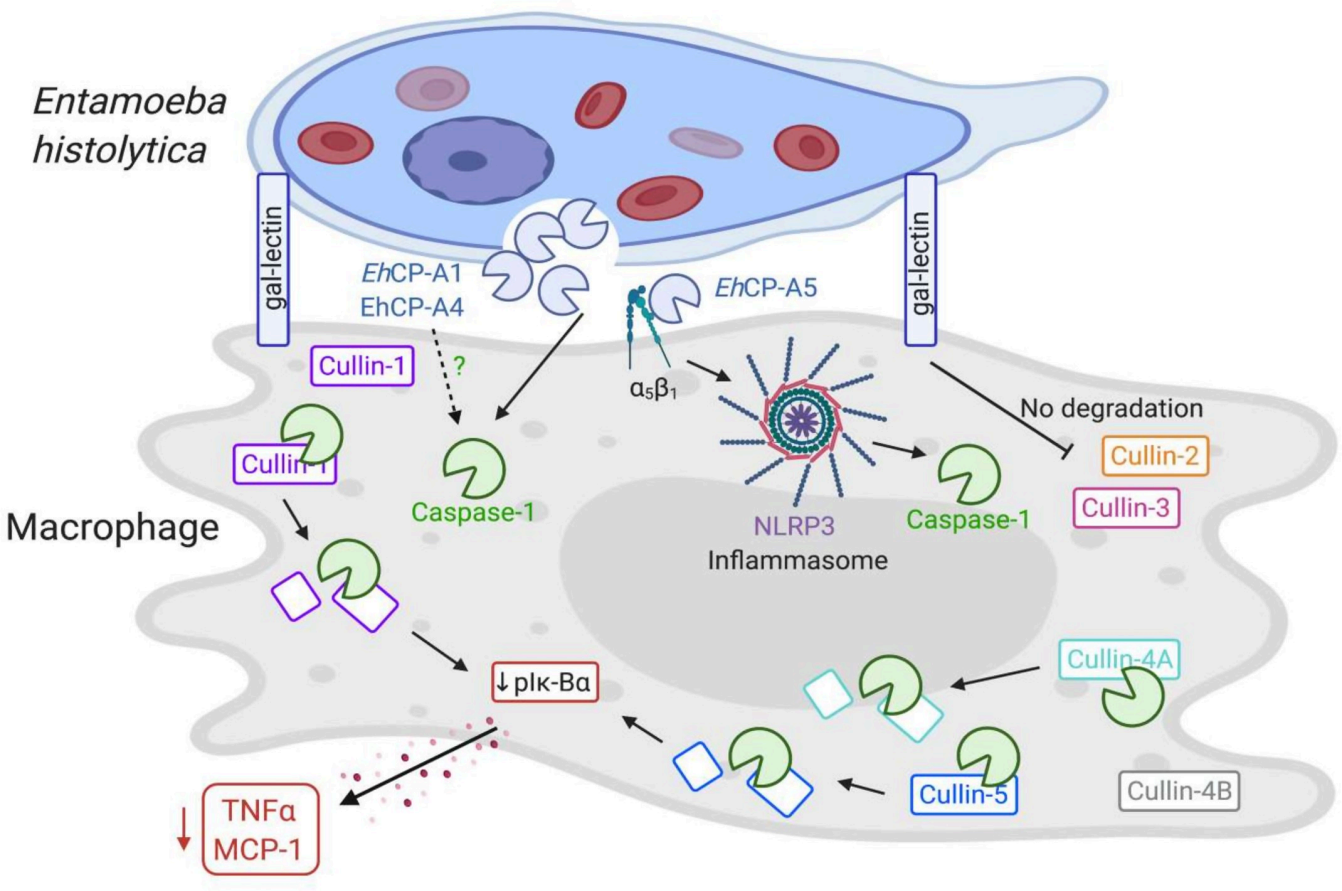
Proposed model of the study. *Eh* Gal-lectin initiates contact with macrophages allowing surface bound *Eh*CP-A5 and vesicle bound cysteine proteinases, *Eh*CP-A1 and *Eh*CP-A4 to interact with macrophage membranes at the intercellular junction. *Eh*CP-A1 and *Eh*CP-A4 degradation of cytoskeletal proteins trigger the NLRP3 inflammasome to activate caspase-1 through an unknown mechanism that subsequently degrades cullin-1/5, that in turn decreased the phosphorylation of Iκ-Bα inhibiting the transcription and secretion of NF-κB dependent pro-inflammatory cytokines. *Eh*-macrophage interaction also led to the degradation of cullin-4A which was partially dependent on caspase-1 but independent in the degradation of cullin-4B. Cullin-2/3 were not degraded in response to *Eh*.

To explore how cullin-1/5 regulates NF-κB-mediated pro-inflammatory cytokine production, cullin-1/5 were silenced by siRNA and stimulated with *Eh* and LPS as a positive control. Silencing *cullin-1*/*5* reduced basal protein expression by 50% (**Fig 7A**) and 80% (**Fig 7B**), respectively. In response to *Eh* stimulation in *cullin-1* silenced cells (**Fig 7C**), there was no significant effect on TNF-α mRNA expression as basal expression was already very low in scrambled siRNA cells. The slight increase in TNF-α mRNA expression in response to *Eh* in cullin-5 scramble siRNA cells was inhibited in *cullin-5* silenced cells (**Fig 7D**). As predicted, in response to the positive control LPS, TNF-α mRNA expression was significantly inhibited in both *cullin-1/5* silenced cells as compared to scramble siRNA controls (**Fig 7C and 7D**). The secretion of two NF-κB-mediated pro-inflammatory cytokines were explored in the supernatant of *cullin-1/5* silenced cells. Even though MCP-1 secretion (**Fig 7E and 7F**) in response to both *Eh* and LPS stimulation was low in scramble siRNA control cells, they were significantly inhibited in *cullin-1/5* silenced cells. Inhibition of TNF-α secretion was even more remarkable, with almost undetectable levels in *Eh* stimulated scramble siRNA cells, but higher levels in response to LPS stimulation that was significantly inhibited in *cullin-1/5* silenced cells (**Fig 7G and 7H**). Under these conditions, NF-κB signaling was independent of NLRP3 inflammasome IL-1β release, as no significant change in IL-1β secretion were noted in *cullin-1* (**S8A Fig**) and *-5* (**S8B Fig**) silenced cells in response to *Eh* stimulation as compared to scramble siRNA control cells. Taken together, these results point to a definite role for cullin-1/5 in regulating NF-κB-dependent cytokine production during *Eh*-macrophage contact as depicted in the diagrammatic model (**Fig 8**).

## Discussion

*Eh* has emerged as a successful pathogen because of its remarkable immune evasion strategies [8,45]. One of these strategies is to subvert host cell signaling pathway by initiating the degradation of host proteins. In this regard, we recently identified that *Eh* potentiates IL-1β release from macrophage by cleaving cytoskeletal associated proteins during *Eh*-macrophage contact [25]. In this study, we unmasked the molecular events by which *Eh* abolishes robust NF-κB mediated pro-inflammatory responses in contact with macrophage. Here we show that *Eh* in contact with macrophages and colonic epithelial cells activated caspase-1 that cleaved cullin-1/5 in a dose- and time-dependent manner that was replicated in a colonic loop model of intestinal amebiasis. Cullin-RING ligases (CRLs) target a range of biological processes, including signal transduction, transcriptional control, cell growth and development [26]. To our knowledge, *Eh* is the first report by a pathogen that targets the degradation of the scaffold proteins cullin-1/4A/4B/5. Many pathogens, including viruses and bacteria, interfere with CRLs to subvert crucial signaling pathways directed for the elimination of the pathogen. Human immune deficiency virus-1 (HIV-1) use different strategies to hijack CRLs for degradation and counteraction of host cell proteins [50]. *Mycobacterium tuberculosis*, the causative agent of tuberculosis, was shown to increase neddylation of cullin-1 from dendritic cells (DC) to evade innate immune defenses via inside-out signaling [51].

Caspase-1 was the first caspase to be identified that cleaves pro-IL-1β and pro-IL-18 into its biologically active forms [52]. Caspase-1 substrates repertoire remains elusive and our data unveil a new role for caspase-1 in degrading cullin-1/5. The molecular mechanism for cullin-1/5 degradation in response to *Eh* was mediated by caspase-1 based on the kinetics of caspase-1 activation with simultaneous cleavage of cullin-1/5, inhibition of caspase-1 enzyme activity with Y-VAD-fmk and inhibitory effects in THP-1 *CASP1* CRISPR/Cas9 KO, THP-1 def *ASC* and *NLRP3* CRISPR/Cas9 KO cells. Culin-2/3 were resistant to degradation during *Eh*-macrophage contact supporting the bioinformatics predictions. However, unlike cullin-4B, the degradation of cullin-4A degradation was partially dependent on caspase-1. Bioinformatics predicted two plausible fragment size of ~27 and ~62 kDa from cullin-1 and ~60 and ~30 kDa fragments from cullin-5 post-caspase-1 cleavage. While the ~60 kDa fragment was observed following *Eh*-macrophage contact we also saw degraded fragments of different molecular weights during cullin-1 cleavage which suggests the involvement of other cleavage sites in cullin-1 or the antibody used were not sensitive in detecting all the fragments. The cullin 1/5 antibody used in this study was the best chosen among those tested that showed degradation of both forms of cullin and identified degraded fragments. Cleavage of cullin-1/5 was dependent on contact between live *Eh* (galactose-inhibitable) and macrophage, but not with soluble *Eh* Gal-lectin, fixed *Eh* or *Eh* lysate. Similarly, the degradation of cullin-4A/B was dependent on live *Eh* and macrophage contact, but not with fixed *Eh* or *Eh* lysate. This suggests that live *Eh*-macrophage contact led to the engagement of multiple receptors triggering downstream signaling molecular events. Gal-lectin stimulation of macrophages alone did cleave cullin-1/5 suggesting the exigent requirement of other pathogen associated virulence factors. Different species of *Entamoeba* has differential expression of cysteine proteinases that play a role in virulence. Non-invasive, *Entamoeba dispar* lacks both *Eh*CP-A1 and *Eh*CP-A5 [13]. In *Eh*, *Eh*CP-A1 is highly transcribed *in vitro* [12,53,54] in comparison to *Eh*CP-A4. Reduced invasion of *Eh* has been shown in human intestinal xenografts in SCID mice when *Eh*CP-A1 was inhibited with WRR483. *Eh*CP-A4 is the most up-regulated CPs in mice during cecal infection [12,55]. Specific cysteine proteinase inhibitors have been designed to block the activity both *in vivo* and *in vitro* [14,56]. *Eh* infected mice showed reduced *Eh* burden and inflammation when administered WRR605 (inhibitor of *Eh*CP-A4) [57]. In our study, cleavage of cullin-1/5 was dependent on *Eh* cysteine proteinases *Eh*CP-A1 and *Eh*CP-A4 but not on *Eh*CP-A5 based on pharmacological inhibition of *Eh* cysteine proteinases and *Eh*CP-A5 deficient parasites and appear to act in a similar fashion to activate caspase-6 that cleaved cytoskeletal proteins [25].

Based on our data, we propose that the interaction of *Eh* with macrophage via the Gal-lectin allowed *Eh*CAP-A1 and *Eh*CP-A4 to interact at the intercellular junction leading to the activation of caspase-1 through an unknown mechanism that cleaves the scaffold proteins cullin-1/5 to abrogate NF-κB dependent signaling and pro-inflammatory cytokine release. In this interaction, the degradation of cullin-4A but not cullin-4B, was partial dependent on caspase-1. Cullin-2/3 was not degraded in response to *Eh* stimulation. NF-κB signaling takes centre stage in inducing pro-inflammatory responses [58] by controlled the NF-κB inducible inhibitor protein, Iκ-Bα. Activation of NF-κB signaling depends on the phosphorylation of Iκ-Bα inducible degradation that free the dimer which subsequently translocate to the nucleus [59]. A major finding in this study was that NLRP3 inflammasome activation upon *Eh*-macrophage contact, potentiate the release of IL-1β by inhibiting NF-κB dependent pro-inflammatory cytokines. While the molecular mechanisms for the activation, processing and release of IL-1β is well known [24,35,43,60], prior to this study, it was not known how *Eh* inhibited NF-κB dependent pro-inflammatory cytokines, a phenomenon noted when *Eh* contact macrophages *in vitro* [61] and *in vivo* in colonic loops [62]. Silencing cullin-1/5 downregulated NF-κB dependent TNF-α and MCP-1 pro-inflammatory cytokine production in response to *Eh* and LPS stimulation. Fbxo21, a component of SCF deficient mice showed impaired c-Jun N-terminal kinase (JNK) and p38 signaling pathway ensuing decreased production of pro-inflammatory cytokines and type I interferon, leading to alleviated antiviral state and enhanced viral replication [63]. Similarly, inhibition of neddylation using the inhibitor MLN4924 resulted in repressed influenza virus replication and pro-inflammatory responses. The repressed state during the infection was due to perturbed CRL/NF-κB axis [64]. In another study, CP77, a host range protein of poxvirus was shown to contain an F-Box-Like domain that suppressed NF-κB activation by TNF-α [65]. The p65 subunit possesses strong transcriptional activation potential of NF-κB [66], and in our study, we observed downregulation of p65 from cullin-1/5 silenced cells despite *Eh*/LPS stimulation. *Eh*-induced caspase-1 enzymatic activity that subsequently cleaved cullin-1/5 appear to be a mechanism that tilt the balance in favor of NLRP3 dominated IL-1β secretion while suppressing NF-κB dependent pro-inflammatory cytokines. Protein-protein interaction showed an intriguing relationship between cullin-1, cullin-5 and NF-κB (Rel). Cullin-1 seem to have a closer relationship to Rel even though both cullin-1/5 had remarkable ability to inhibit NF-κB signaling. The variable results obtained with cullin-1/5 siRNA as opposed to caspase-1 mediated degradation to both proteins, suggests that they interact together in the complex to regulate NF-κB signaling. Unfortunately, we could not manipulate both proteins together as it would have provided a better understanding of the dominating effect of one protein over the other. Global Cul-1 knockout mice embryos are lethal [67] that is why we silenced *cullin-1*/*5* genes separately to quantify their effects on NF-κB signaling.

In conclusion, our study revealed that cullin-1/5 are two new proteolytic targets for inflammatory caspase-1 during *Eh*-macrophage contact that inhibits NF-κB signaling. We recently reported a global proteomic study using BMDM stimulated with *Eh* for 10 min that showed an interesting pattern of modulation of host proteins [68]. Our data revealed 3 unique proteins: COP9 signalosome complex subunit 5, cullin-associated nedd8-dissociated protein 1 (CAND 1) and nedd8 ultimate buster protein 1, which were downregulated from *Eh* stimulated BMDM. CAND 1 binding to unneddylated cullin-Rbx1 complex occludes the SCF complex assembly [69]. The downregulation of these proteins’ points toward disruption of the CRL complex leading to aborted NF-κB signaling. Taken together these findings reveal a new molecular dimension on how *Eh* abrogates NF-κB signaling from macrophage by targeting cullins.

## Materials and methods

### E. histolytica culturing

*Eh* HM-1:IMSS, virulent strain of *Eh* were grown axenically in TYI-S-33 medium supplemented with 100 U/ml penicillin and 100μg/ml streptomycin sulfate at 37°C in borosilicate glass tube as described earlier [70]. Post 72 h (log phase), *Eh* were harvested by keeping on ice for 9 min followed by centrifugation at 200×g for 5 min at 4°C. Following centrifugation, *Eh* was resuspended in RPMI and adjusted to 1×10^6^ cells/ml. *Eh*CP-A5 deficient parasites [18] was a gift from D. Mirelman that plays a role in disease pathogenesis.

### Cell culture

THP-1 monocytic cells were obtained from ATCC. It was maintained in RPMI media supplemented with 10mM HEPES, 10% Fetal bovine serum (FBS), 50μM 2-mercaptoethanol, and antibiotics (100 U/ml penicillin and 100μg/ml streptomycin sulfate) in a 37°C incubator with 5% CO_2_ level. THP-1 monocytic cells were differentiated into Mφ using 50ng/ml phorbol-12-myristate-13-acetate (PMA) following overnight incubation. For experiments, 8×10^5^ cells/well were seeded in 12 well plate with complete media. Human T84 epithelial cells (ATCC) were cultured in Dulbecco’s modified Eagle’s medium F12 (1:1) supplemented with 10% FBS and antibiotics (100 U/ml penicillin and 100μg/ml streptomycin sulfate) in an incubator maintained at 37°C with 5% CO_2_ level. For experiments, 3x10^5^ cells/well were seeded in 12 well plate and grown to 80% confluency. For mouse bone marrow derived macrophages (BMDMs), cells were cultured in complete medium complemented with 30% L-929 supernatants for 6 days. For *Eh* stimulations, cells were stimulated at a 10:1 ratio of macrophage to *Eh* for the indicated dose and time at 37°C in serum free RPMI (400 μl/well). Post-incubation, cells were washed with ice cold PBS and cell lysates were prepared using cell lysis buffer (20mM Tris, 1% Triton X-100, 1mM EDTA, 100mM NaCl, 200mM orthovanadate, sodium fluoride, phenylmethanesulfonyl fluoride, 0.1% sodium dodecyl sulfate, aprotinin, leupeptin, and protease inhibitor cocktail). For the inhibitor experiments, differentiated THP-1 cells were pre-incubated with the indicated inhibitors for 30 min in serum free RPMI at 37°C prior to *Eh* stimulation.

### *CASP1*-KO-THP-1, *ASC* def THP-1 KO and *NLRP3* KO cells

THP-1 def *ASC* were procured from Invivogen (thp-dasc). *CASP-1* CRISPR/Cas9 KO THP-1 cell was a gift from V. Hornung (Institute of Molecular Medicine, University Hospital, University of Bonn, Germany) and verified for CASP1. For *CASP-1* CRISPR/Cas9 KO THP-1 cell, plasmid encoding the CMV-mCherry-CAS9 cassette and a gRNA under the U6 promoter was used. *NLRP3* CRISPR/Cas9 KO THP-1 cell line was a kind gift from Dr. Muruve (Department of immunology, University of Calgary, Canada). Cells were maintained as described above.

### Western blotting

Post stimulations, cells were washed with ice-cold PBS and lysed in cell lysis buffer. The lysed cell suspension was then centrifuged at 12000g for 5 min at 4°C. The supernatant was harvested and 20μg of cytoplasmic extracts was loaded onto 7.5% SDS- polyacrylamide gel electrophoresis PAGE and subsequently transferred onto nitrocellulose membrane. The blotted membranes with transferred proteins were subjected to blocking with 5% skim milk for 1h at room temperature (RT). The membrane was incubated overnight with specific primary antibodies at 4°C followed by probing with horse radish peroxidase (HRP) labeled secondary antibodies. The blots were developed by chemiluminescence using either SuperSignal Chemiluminescence Reagents (Pierce Biotechnology) or ChemiLucent ECL detection (EMD Millipore). Primary antibodies used were anti -cullin-1 (Thermo Fisher scientific, 71–8700), anti-cullin-5 antibody (Novus Biologicals, NBP1-22970), anti-cullin-1 antibody (Cell Signaling Technology, 4995), anti-cullin-2 antibody (Thermo Fisher scientific, 700179), anti-cullin-3 antibody (Thermo Fisher scientific, PA5-17397), anti-cullin-4A antibody (Thermo Fisher scientific, PA5-85573), anti-cullin-4B antibody (Thermo Fisher scientific, PA5-51084), anti-p-IκBα (Cell Signaling Technology, 2859), anti-IκBα (Cell Signaling Technology, 4812), anti-GAPDH (Millipore, sigma), anti-NF-κB p65 (Santa Cruz Biotechnology, sc-8008), anti-Caspase-1 antibody (Santa Cruz Biotechnology, sc-622) and anti-Nedd8 antibody (Cell Signaling Technology, 2745). It is pertinent to mention here that the immunoblot of cullin-1 appears as a doublet at ~89 kDa. The lower band (~85 kDa) corresponds to cullin-1 while the upper band (~94 kDa) corresponds to neddylated cullin-1. Thermo Fisher antibody for cullin-1 also detects an additional band at ~68 kDa of unknown identity. Due to its high immunoreactivity in detecting the cleaved fragments, this specific antibody was used for the experiments. The cullin-5 antibody detects doublet at around ~90 kDa (lower band corresponds to cullin-5) and upper band at ~99 kDa corresponds to neddylated cullin-5.

### Immunoprecipitation

Anti-cullin-1 (Thermo Fisher scientific, 71–8700), and anti-cullin-5 antibody was used to pull down cullin-1/5 from cell lysates (200μg). 10% A/G beads (Santa Cruz) were used at 4°C overnight. Complexes of protein-bead were washed 3 times and were subjected to boiling at 95°C for 5 min. Immunoblotting was done using anti-cullin-1/5 antibody.

### In vitro enzymatic assays

Recombinant cullin-1 (ab 131835) was procured from Abcam, and recombinant caspase-1 (ALX-201-056) was purchased from Enzo life sciences. For experiments, THP-1 cells were lysed as described above, and 200μg of cell lysate was incubated with anti-cullin-1 antibody (1:50) and anti-cullin-5 antibody (1:50) for 4h and with Protein A/G plus agarose beads for 1.5h. Post immunoprecipitation, complexes of beads-protein were incubated with recombinant caspase-1 (2 units) enzyme either for 4h or overnight at 37°C. Immunoprecipitates were subjected to boiling at 95°C for 5 min before loading onto SDS-PAGE gel. Recombinant cullin-1 (50 ng) and recombinant caspase-1 enzyme (2 units) were incubated together at 37°C overnight and were subjected to boiling at 95°C for 5 min before loading onto SDS-PAGE gel. Immunoprecipitates were also subjected to silver staining as per the manufacturer’s protocol (Pierce Silver Stain kit, # 24612).

### Colonic loop studies

C57BL/6 mice from Charles River were used for the colonic loop studies inoculated with log-phase virulent *Eh* suspended in 100μL PBS (1×10^6^) as described previously [71]. Briefly, following laparotomy, the colon was externalized and ligation with suture was done at distal and proximal ends with utmost care to keep blood vessels, nerves, and mesenteries intact. Colonic loop is considered as a short-term infection model. Post 3h, loops were excised, and tissues were used for protein isolation and western blotting.

### Cullin-1 and cullin-5 siRNA

THP-1 derived Mφ were electroporated (Amaxa biosystems Nucleofector II) with 100nM of Cullin-1/5 siRNA respectively and scramble siRNA was used as a control (Dharmacon, On target plus SMART pool siRNA) using Mirus Ingenio solution (MIR 50114) following the manufacturer’s protocol. 100μl of mirus solution was used for each transfection. Every 24 h media was replaced with new complete media. Post 72 h following transfection, cells were stimulated with *Eh*. Human CUL-1 siRNA (Dharmacon, 8454) pool contains the following target sequence: CGACAGCACU-CAAAUUAAA, GGUUAUAUCAGUUGUCUA, AGACUUGGAUUUCAG-CAUU, CAACGAAGAGUUCAGGUUU and Human CUL-5 siRNA (Dharmacon, 8065) pool contains following target sequence: GACACGACGUCUUAUAUUA, CGUCUAAUCUGUU-AAAGAA, GAUGAUACGGCUUUGCUAA, GUUCAACUACGAAUACUA.

### Real-time PCR

Total RNA was extracted using the E.Z.N.A. Total RNA Kit (Omega Bio TEK) from cells. The yield and purity of the extracted RNA was measured by the A260/A280 ratio (NanoDrop, Thermo Scientific). qScript cDNA Synthesis kit was used for making the cDNA (Complementary DNA). Rotor Gene 3000 Real- time PCR system (Corbett Research) was used for performing the Real-time quantitative PCR. Briefly, the reaction mixture contained cDNA (1:10 dilution), SYBR Green PCR Master Mix (Qiagen) and 1μM of primers (Forward+ Reverse). 2^-ΔΔCT^ equation was used to analyze the results and was expressed as relative m-RNA expression. The following primer sequence were used; forward: 5′-AAGCCTGTAGCCCATGTTGT-3′ and reverse: 5′-GAGGTA-CAGGCCCTCTGATG-3′ for TNF-α and forward: 5′-GGATTTGGTCGTATTGGG-3′ and reverse: 5′-GGAAGATGGTGATGGGATT-3′ for GAPDH.

### Human focused 15-plex cytokine array

Supernatants from THP-1 macrophage silenced for cullin-1/5 (stimulated with *Eh* and/or LPS) were analyzed for the presence of cytokines and chemokines using the human cytokine array pro-inflammatory focused-15-plex (HDF15) discovery assay (Eve Technologies, Calgary, AB, Canada). The following cytokines were measured: GM-CSF, IL-10, IL-8, IL-6, MCP-1, IL-12p40, IL-12p70, IL-2, IL-4, TNF-α, IFN-γ, IL-1β, IL-13 and IL-5, IL-1Ra.

### Bioinformatics analysis

Software Peptide Cutter (ExPASy) (https://web.expasy.org/peptide_cutter/) that predicts potential cleavage sites by different chemicals and proteases against a given query (Protein sequence) was used for determining the potential cleavage site in different cullin proteins. For the protein-protein interaction study STRING v11 (https://string-db.org) software was used.

### Statistics

Graphpad Prism 7 (Graph-Pad Software, San Diego, CA) was used for all the statistical analysis. Student’s t test was used when two groups were compared. One-way analysis of variance (ANOVA) followed by post hoc Bonferroni test was used when two or more groups were compared. Statistical significance was assumed at p < 0.05. Image J software was used for densitometric analysis of western blots.

## Supporting information

S1 Fig *Eh* promotes global neddylation of host cell proteins during *Eh*-macrophage contact. **(A)** THP-1 macrophages were incubated with different *Eh*: macrophage ratios and for different time points as indicated. Post incubation, cells were washed and lysed and immunoblotted with anti-cullin-1 antibody (Cell Signaling Technology # 4995). **(B, C)** THP-1 macrophages were incubated with with two different concentrations (400nM and 500nM) of the neddylation inhibitor, MLN4924 for 2h. Post incubation, cells were washed and lysed and immunoblotted with anti-cullin-1 and-5 antibody. **(D)** THP-1 macrophages were incubated with different dose and time with *Eh* as indicated in Fig 1B. Post incubation, cells were washed and lysed and immunoblotted with anti-Nedd8 antibody.
(TIFF)

S2 Fig *Eh* promotes the degradation of cullin-1, cullin-5, cullin-4A and cullin-4B from macrophages. THP-1 macrophages were incubated with live *Eh*: macrophage ratio (1:10) for 10 min. Post incubation, cells were washed and lysed and immunoblotted with **(A)** anti-cullin-1 antibody. **(B)** anti-cullin-5 antibody **(C)** anti-cullin-4A antibody **(D)** anti-cullin-4B antibody **(E)** anti-cullin-2 antibody **(F)** anti-cullin-3 antibody. Data are representative of two different experiments.
(TIFF)

S3 Fig *Eh* promotes the degradation of neddylated and unneddylated cullin-1/5. **(A-D)** Histograms of densitometric analysis of the western blots in **Fig 1B, C, D and E**, respectively. Data are representative of three independent experiments. Bar represent mean ± SEM. * P <0.05, **P<0.01, and ***P<0.001. **(E-F)** Histogram of densitometric analysis of the western blots in **Fig 1H and I.**
(TIFF)

S4 Fig *Entamoeba histolytica* promotes the degradation of the cullin-1/5 from BMDM in a time and dose-dependent manner. BMDM were incubated with different *Eh*: macrophage ratios or time and the degradation of cullin-1 (**A, B**) and cullin-5 (**C, D)** determined. Post incubation, cells were washed and lysed in cell lysis buffer and equal amounts of protein was loaded on to SDS-PAGE gels (7.5%) and immunoblotted against the indicated antibody. Highlighted boxed areas on the figures show point of interest for cullin-1/5 as described in text. Data are representative of two independent experiment.
(TIFF)

S5 Fig Cullin-1/5/4A/4B degradation is dependent on live *Eh* in contact with macrophages. THP-1 macrophages were incubated with live *Eh*: macrophage ratio (1:10), fixed *Eh*: macrophage ratio (1:10) for 10 min and with equivalent amount of freeze thawed *Eh* cell lysate. Live *Eh* were fixed with 1.5% glutaraldehyde for 1h at 4°C and washed twice with sterile cold PBS before use. Post incubation, the supernatant (SN) was TCA precipitated and equal amounts was loaded onto the SDS-PAGE gel to enumerate caspase-1 activation with anti-caspase-1 antibody, while the cell lysates were immunoblotted with the anti-cullin-1/5/4A/4B and anti-GAPDH antibody. Data are representative of two different experiments.
(TIFF)

S6 Fig Cullin1/5/4A degradation is mediated by caspase-1 during *Eh*-macrophage contact. **(A)** Histogram corresponds to **Fig 5A**, **(B)** Histogram of densitometric analysis for **Fig 5B**, **(C)** for **Fig 5C** and **(D)** for **Fig 5D**. Data are representative of densitometric analysis of three independent experiments. Bars represent mean ± SEM. * P <0.05, **P<0.01, and ***P<0.001. ns = not significant. THP-1 macrophages were pre-incubated with the pan-caspase inhibitor Z-VAD-fmk (100μM) and caspase-1 specific inhibitor Z-YVAD-fmk (100μM) for 1 h followed by stimulation with *Eh*: macrophage ratio (1:10) for 10 min and Wild type (WT) THP-1 and *CASP1* CRISPR/Cas9-KO macrophages were stimulated with *Eh*: macrophage ration (1:10) for 10 min. Post incubation, cells were washed and lysed in cell lysis buffer and equal amounts of protein was loaded on to SDS-PAGE gels (7.5%) and immunoblotted against the **(E)** anti-cullin-4A antibody and **(F)** anti-cullin-4B antibody. Data are representative of two different experiments.
(TIFF)

S7 Fig Caspase-1 activation with concomitant degradation of cullin-1/5. **(A)**THP-1 macrophages were incubated with *Eh* (10:1 ratio) for increasing time (5 to 30 min) and or /with the pan-caspase inhibitor Z-VAD-fmk (100μM) and caspase-1 specific inhibitor Z-YVAD-fmk (100μM) for 1 h followed by stimulation with *Eh* (10:1 ratio) for 10–30 min. LPS + nigericin (LPS 100 ng/ml, nigericin 10 μM) was used as a positive control for NLRP3 inflammasome activation of caspase-1. Post incubation, the supernatant (SN) was TCA precipitated and equal amounts was loaded onto the SDS-PAGE gel to enumerate caspase-1 activation with anti-caspase-1 antibody, while the cell lysates were immunoblotted with the anti-cullin-1, anti-cullin-5 and anti-GAPDH antibody. Highlighted boxed areas on the figures show point of interest for cullin-1/5 as described in text. **(B)** Immunoprecipitated cullin-1 was incubated with recombinant caspase-1 overnight and was loaded onto SDS-PAGE and silver stained to determine cullin degradation.
(TIFF)

S8 Fig IL-1β release during *Eh*-macrophage contact was independent of cullin-1/5. **(A, B)** THP-1 macrophages silenced for cullin-1/5 siRNA was incubated with *Eh* or LPS for 2h and pro-inflammatory cytokine (IL-1β) levels measured using human cytokine array pro-inflammatory focused 15-plex discovery assay. Data are representative of three independent experiments and statistical significance was carried out with Student t test.
(TIFF)
