# Supplementary figures and images for: Entamoeba histolytica activation of caspase-1 degrades cullin that attenuates NF-κB dependent signaling from macrophages
Source: PLoS Pathog. 2021 Sep 9;17(9):e1009936. doi: 10.1371/journal.ppat.1009936 (PMC8454965; doi:10.1371/journal.ppat.1009936)

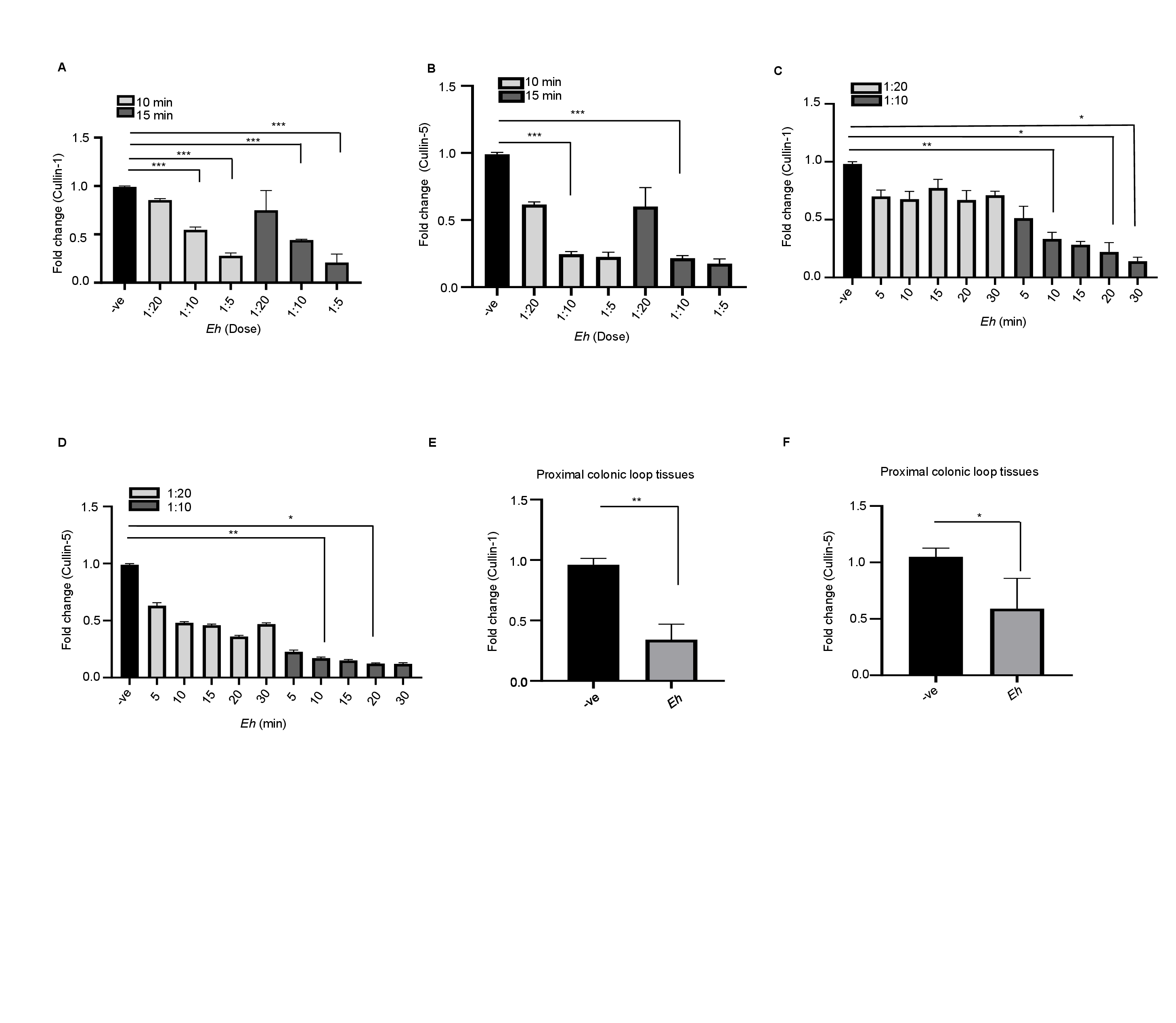

Supplement: S3 Fig — (A-D) Histograms of densitometric analysis of the western blots in Fig 1B, C, D and E, respectively. Data are representative of three independent experiments. Bar represent mean ± SEM. * P <0.05, **P<0.01, and ***P<0.001. (E-F) Histogram of densitometric analysis of the western blots in Fig 1H and I. (TIFF) [file ppat.1009936.s003.tiff]

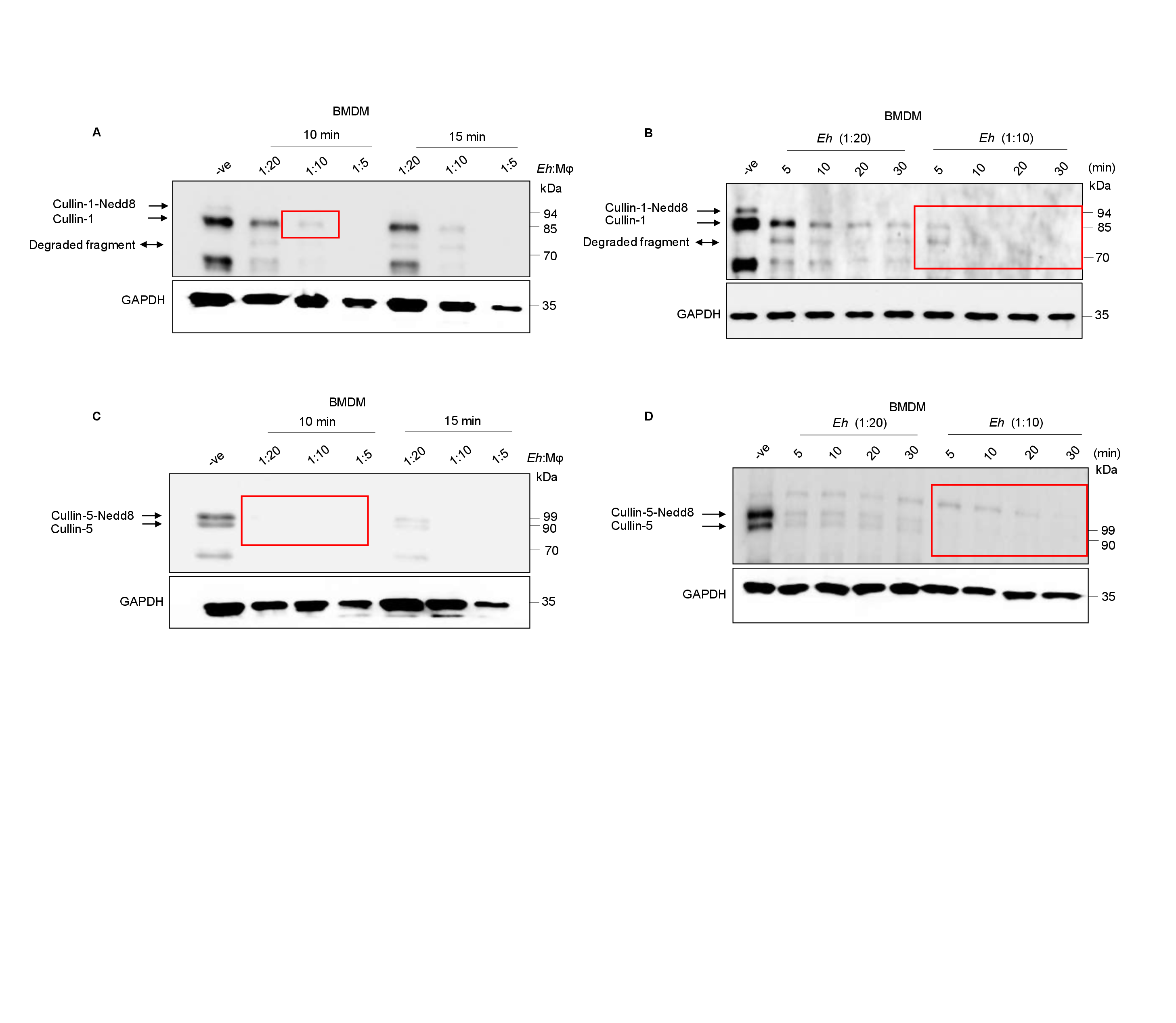

Supplement: S4 Fig — BMDM were incubated with different Eh: macrophage ratios or time and the degradation of cullin-1 (A, B) and cullin-5 (C, D) determined. Post incubation, cells were washed and lysed in cell lysis buffer and equal amounts of protein was loaded on to SDS-PAGE gels (7.5%) and immunoblotted against the indicated antibody. Highlighted boxed areas on the figures show point of interest for cullin-1/5 as described in text. Data are representative of two independent experiment. (TIFF) [file ppat.1009936.s004.tiff]

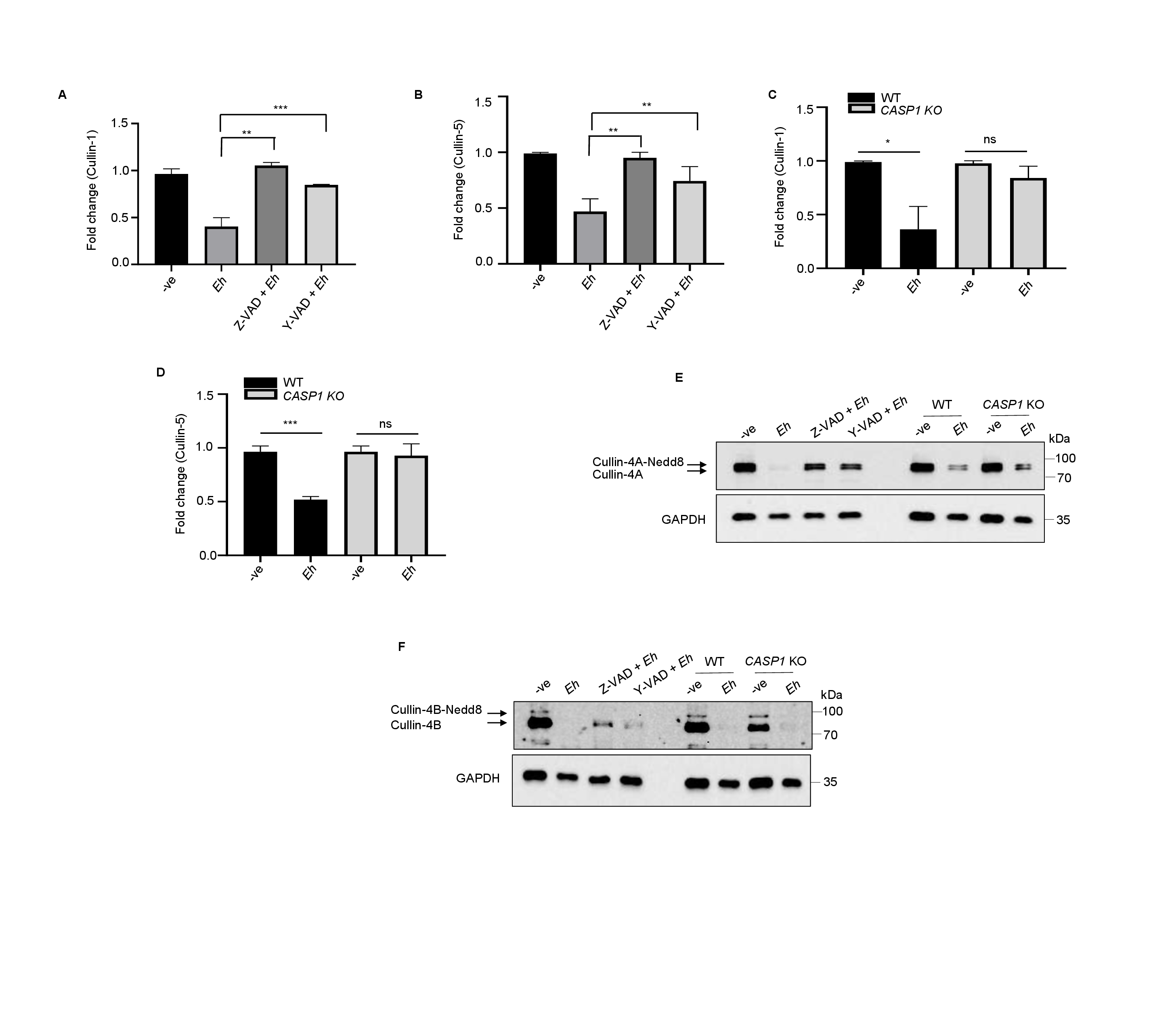

Supplement: S6 Fig — (A) Histogram corresponds to Fig 5A, (B) Histogram of densitometric analysis for Fig 5B, (C) for Fig 5C and (D) for Fig 5D. Data are representative of densitometric analysis of three independent experiments. Bars represent mean ± SEM. * P <0.05, **P<0.01, and ***P<0.001. ns = not significant. THP-1 macrophages were pre-incubated with the pan-caspase inhibitor Z-VAD-fmk (100μM) and caspase-1 specific inhibitor Z-YVAD-fmk (100μM) for 1 h followed by stimulation with Eh: macrophage ratio (1:10) for 10 min and Wild type (WT) THP-1 and CASP1 CRISPR/Cas9-KO macrophages were stimulated with Eh: macrophage ration (1:10) for 10 min. Post incubation, cells were washed and lysed in cell lysis buffer and equal amounts of protein was loaded on to SDS-PAGE gels (7.5%) and immunoblotted against the (E) anti-cullin-4A antibody and (F) anti-cullin-4B antibody. Data are representative of two different experiments. (TIFF) [file ppat.1009936.s006.tiff]

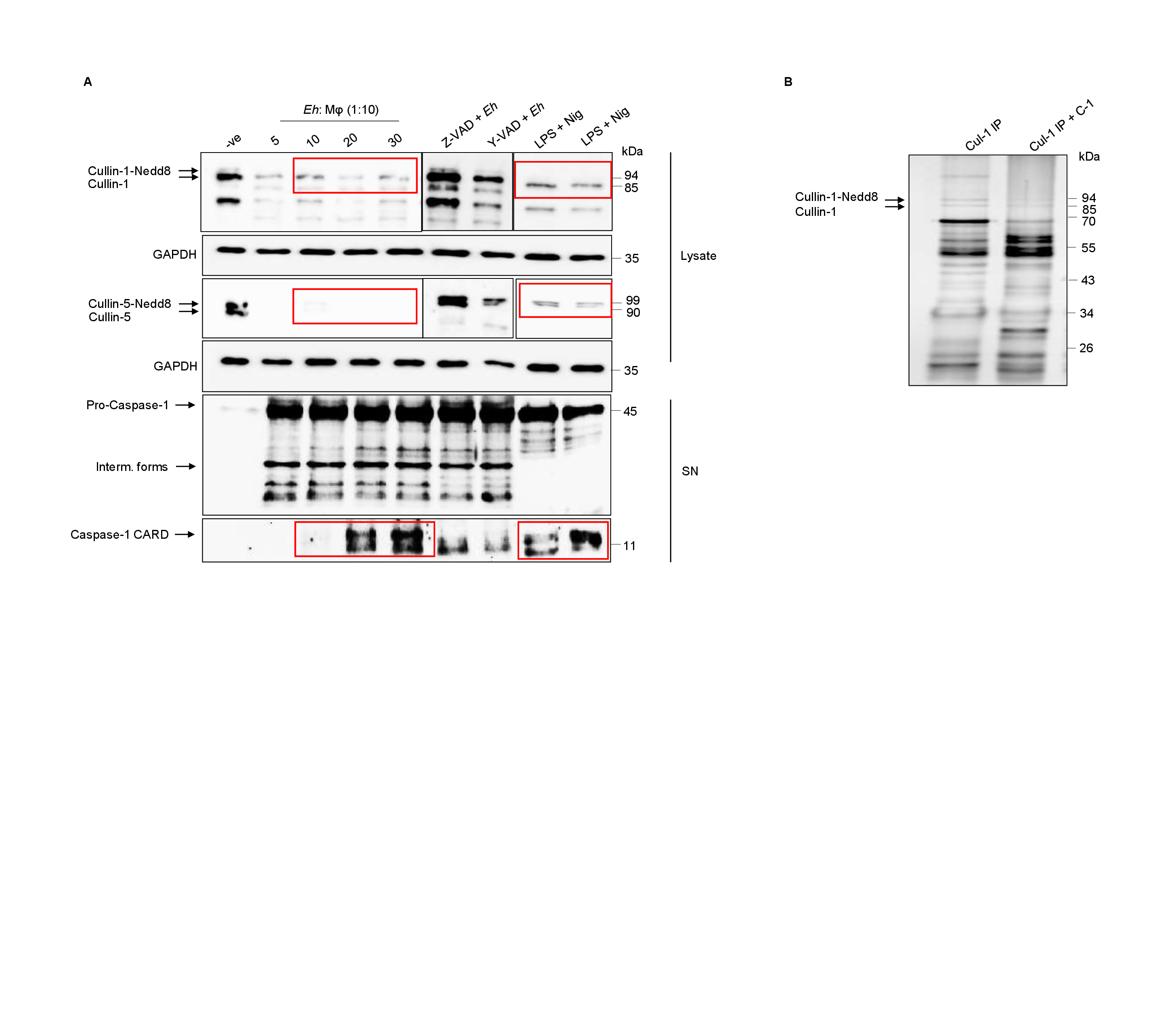

Supplement: S7 Fig — (A)THP-1 macrophages were incubated with Eh (10:1 ratio) for increasing time (5 to 30 min) and or /with the pan-caspase inhibitor Z-VAD-fmk (100μM) and caspase-1 specific inhibitor Z-YVAD-fmk (100μM) for 1 h followed by stimulation with Eh (10:1 ratio) for 10–30 min. LPS + nigericin (LPS 100 ng/ml, nigericin 10 μM) was used as a positive control for NLRP3 inflammasome activation of caspase-1. Post incubation, the supernatant (SN) was TCA precipitated and equal amounts was loaded onto the SDS-PAGE gel to enumerate caspase-1 activation with anti-caspase-1 antibody, while the cell lysates were immunoblotted with the anti-cullin-1, anti-cullin-5 and anti-GAPDH antibody. Highlighted boxed areas on the figures show point of interest for cullin-1/5 as described in text. (B) Immunoprecipitated cullin-1 was incubated with recombinant caspase-1 overnight and was loaded onto SDS-PAGE and silver stained to determine cullin degradation. (TIFF) [file ppat.1009936.s007.tiff]

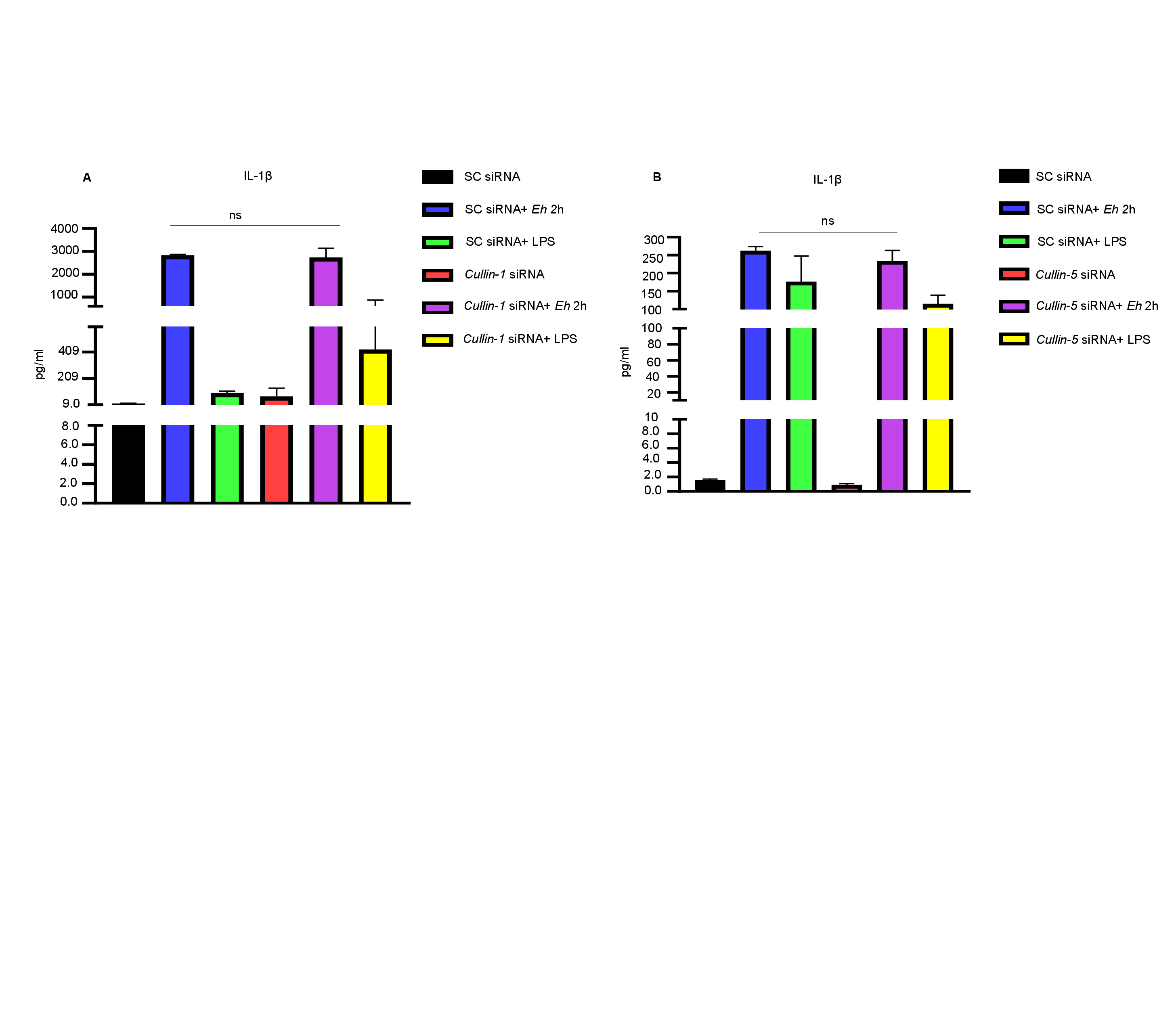

Supplement: S8 Fig — (A, B) THP-1 macrophages silenced for cullin-1/5 siRNA was incubated with Eh or LPS for 2h and pro-inflammatory cytokine (IL-1β) levels measured using human cytokine array pro-inflammatory focused 15-plex discovery assay. Data are representative of three independent experiments and statistical significance was carried out with Student t test. (TIFF) [file ppat.1009936.s008.tiff]
